# Supplementary material for: Synthesis and Insecticidal Activity of Novel Anthranilic Diamide Insecticides Containing Indane and Its Analogs
Source: Int J Mol Sci. 2024 Feb 19;25(4):2445. doi: 10.3390/ijms25042445 (PMC10889706; doi:10.3390/ijms25042445)
Supplement: Supplementary file 1 [file ijms-25-02445-s001.zip › ijms-2836422-supplementary.pdf]

Synthesis and insecticidal activity of novel anthranilic diamide insecticides containing indane and its analogs

Zhi-hui Yang, Rui-han Hu, Jing-jing Chen, Xiao-hua Du*

Catalytic Hydrogenation Research Center, Zhejiang Key Laboratory of Green Pesticides and Cleaner Production Technology, Zhejiang Green Pesticide Collaborative Innovation Center, Zhejiang University of Technology, Hangzhou 310014, P. R. China

*Corresponding author.
E-mail addresses: duxiaohua@zjut.edu.cn

Supporting Information

Contents:

1. ^{1}H NMR and ^{13}C NMR spectra of target compounds **8a–8u;**

2. Safety assessment data of compound **8q** and chlorantraniliprole.

1. ^{1}H NMR and ^{13}C NMR spectra of target compounds 8a–8u.

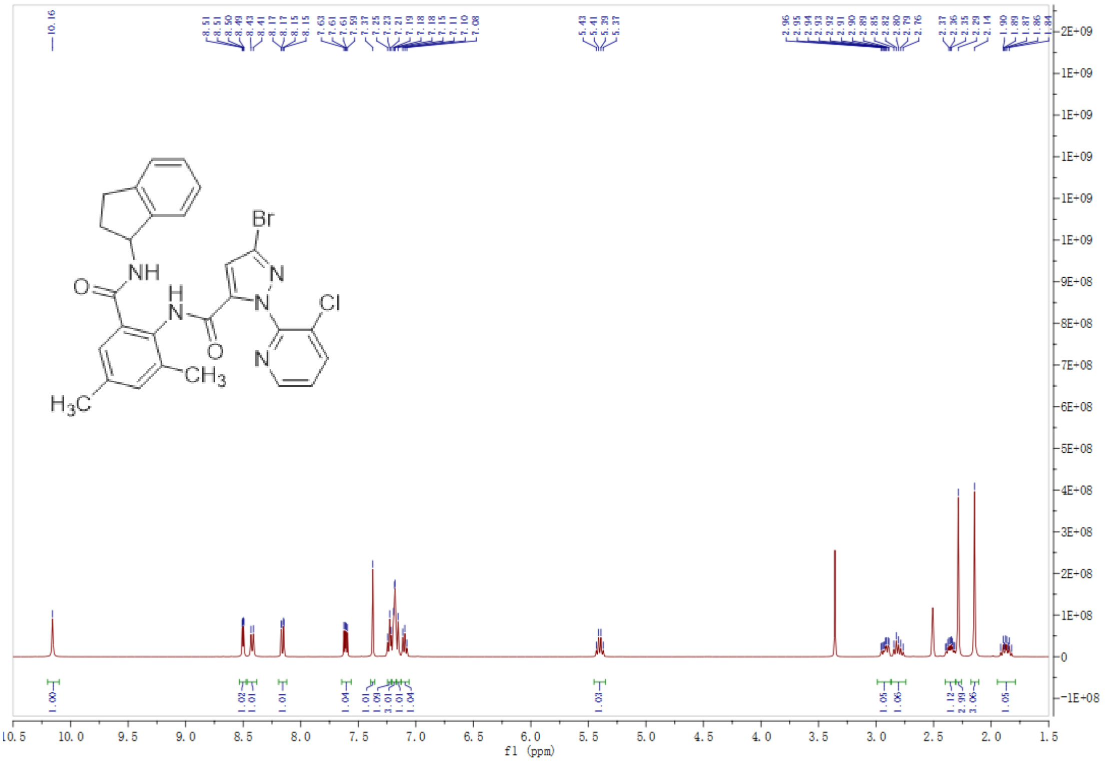

Figure S1. The ^{1}H NMR spectrum of **8a** (DMSO-d_6).

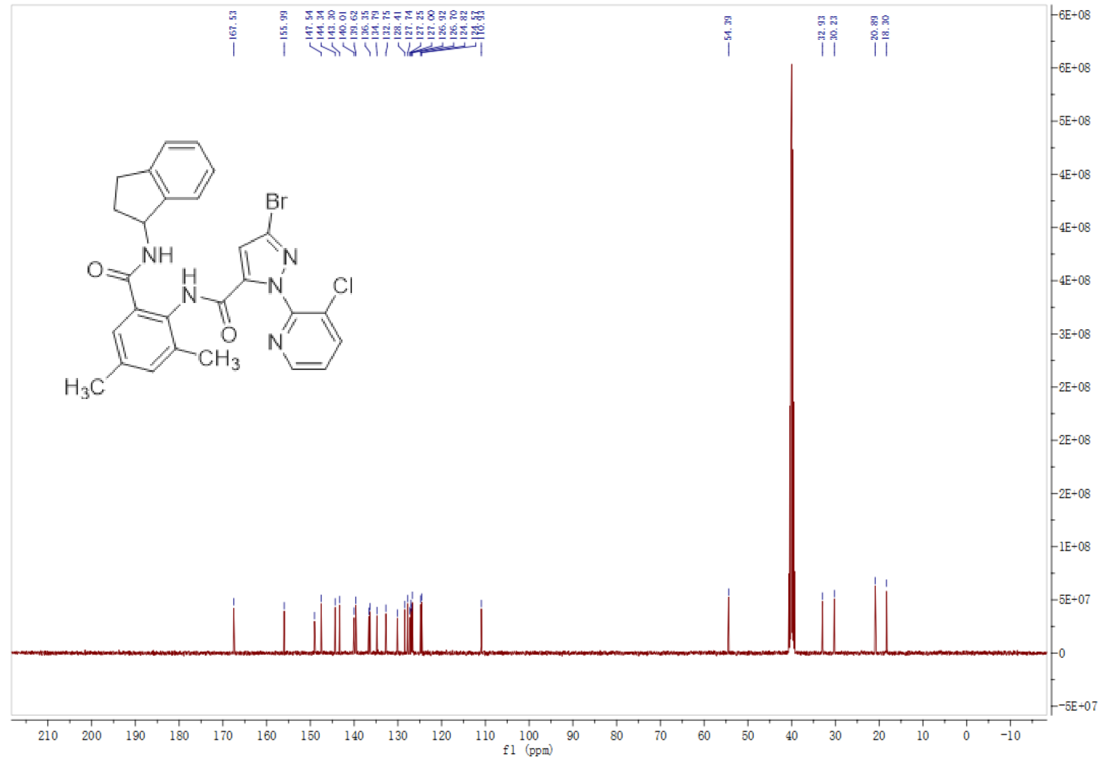

Figure S2. The ^{13}C NMR spectrum of **8a** (DMSO-d_6).

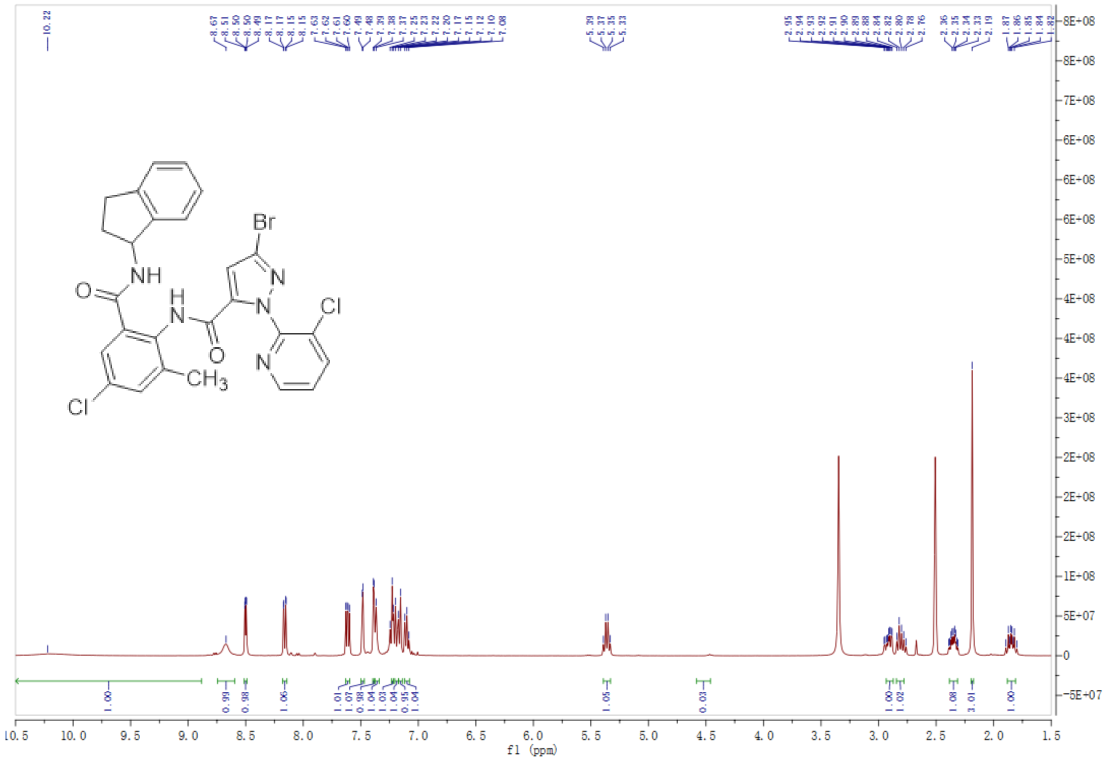

Figure S3. The ¹H NMR spectrum of **8b** (DMSO-d_6).

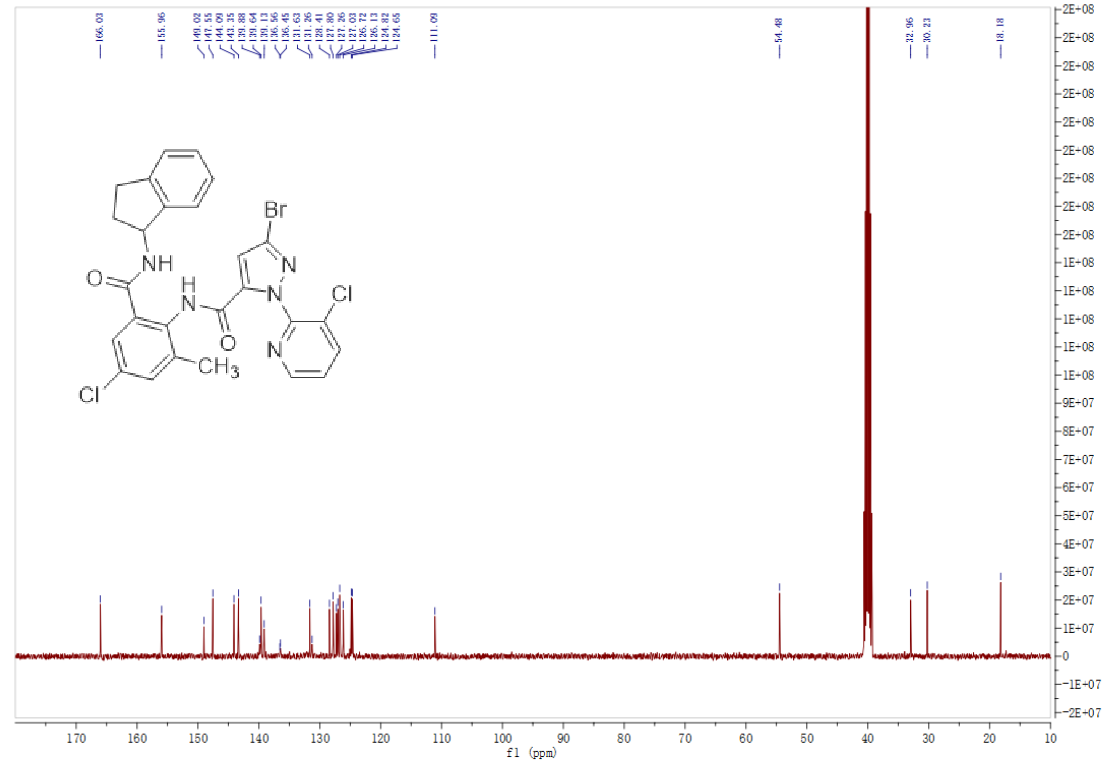

Figure S4. The ¹³C NMR spectrum of **8b** (DMSO-d_6).

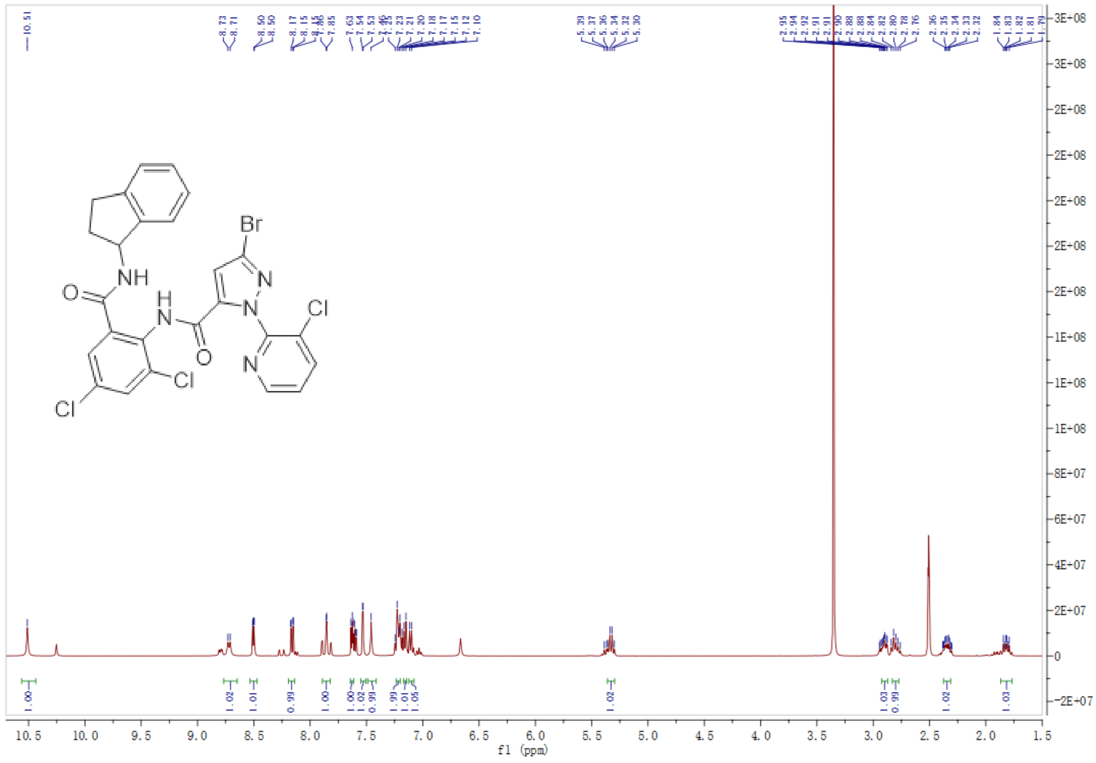

Figure S5. The ¹H NMR spectrum of **8c** (DMSO-d_6).

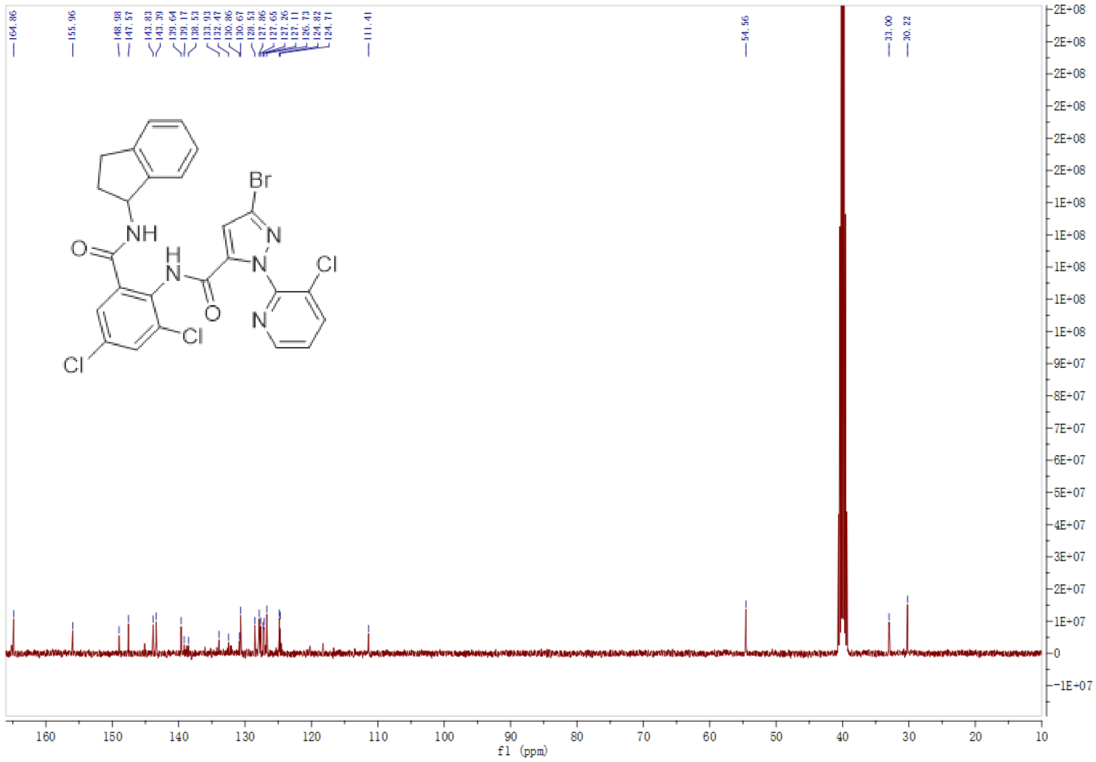

Figure S6. The ¹³C NMR spectrum of **8c** (DMSO-d_6).

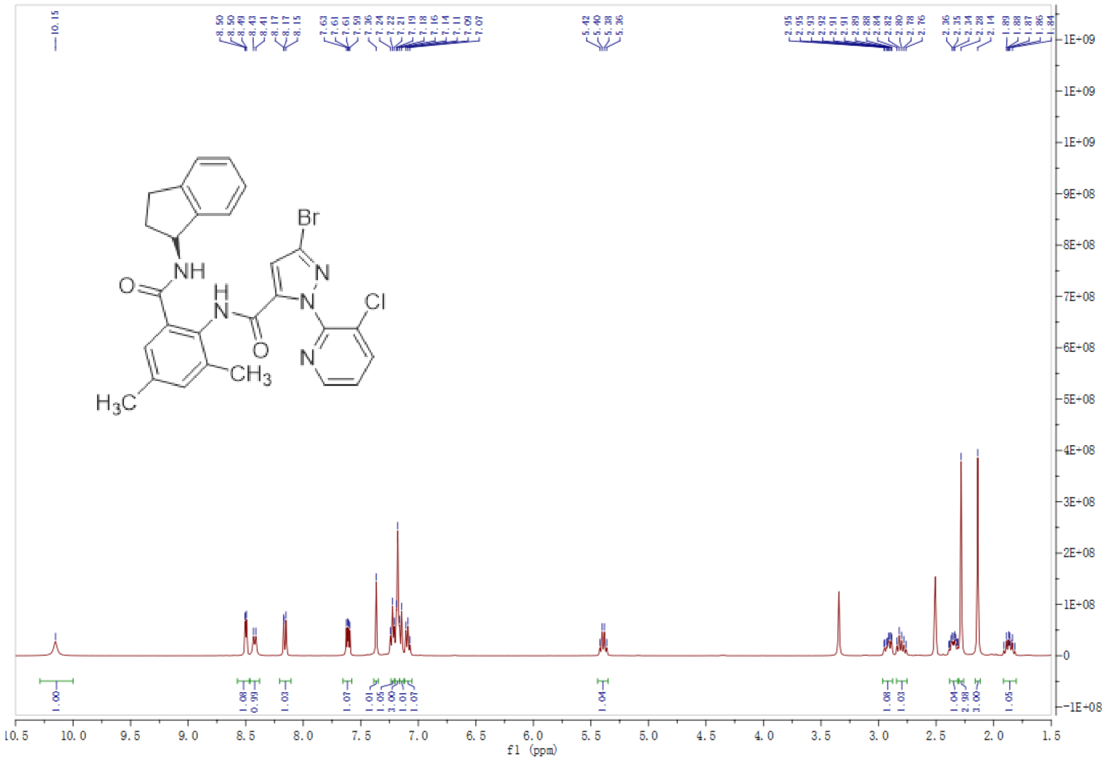

Figure S7. The ¹H NMR spectrum of **8d** (DMSO-d_6).

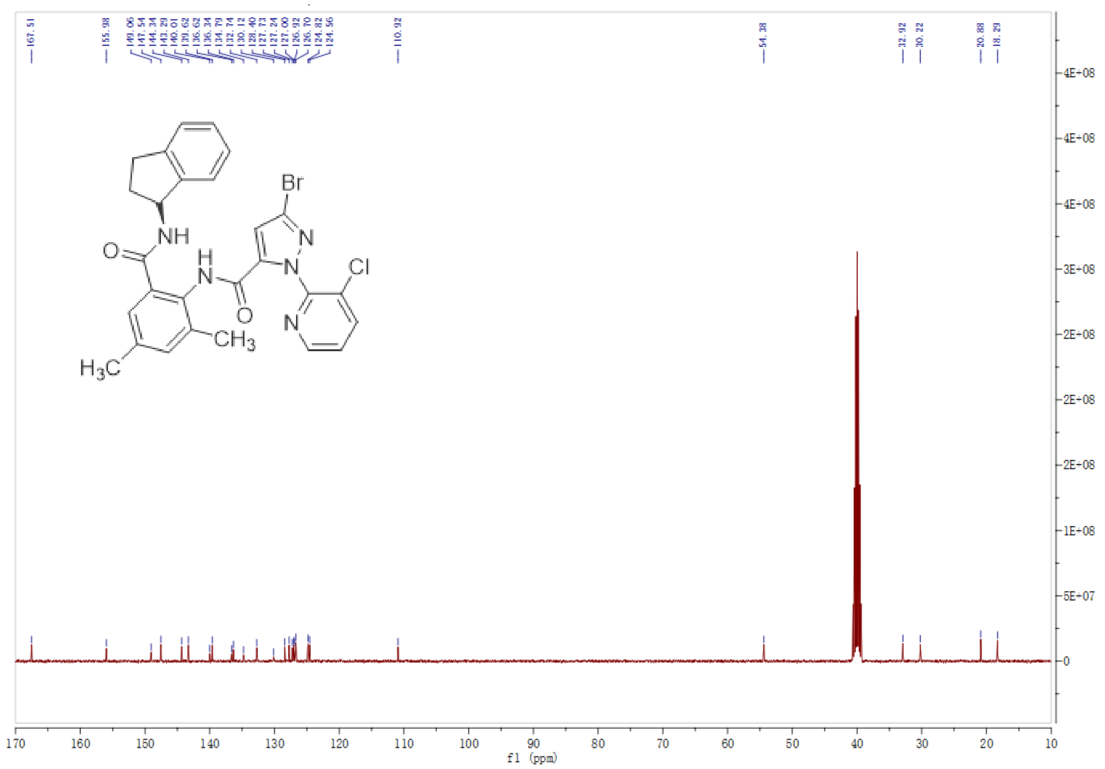

Figure S8. The ¹³C NMR spectrum of **8d** (DMSO-d_6).

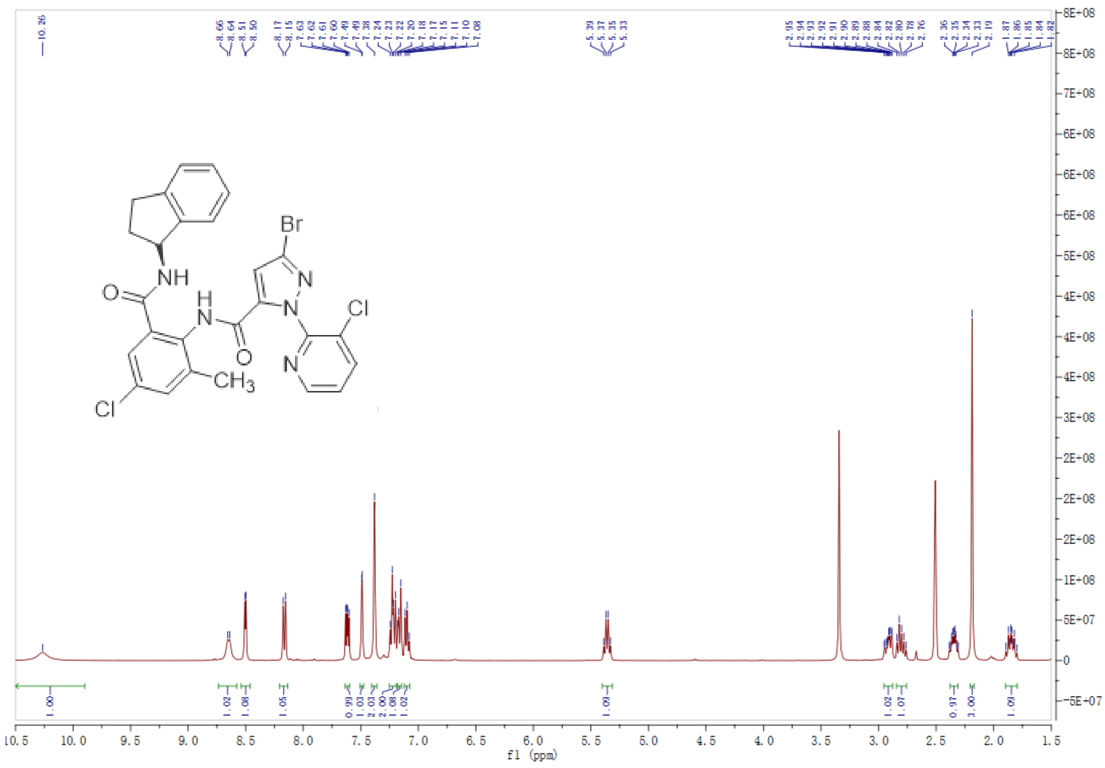

Figure S9. The ^{1}H NMR spectrum of **8e** (DMSO-*d*6).

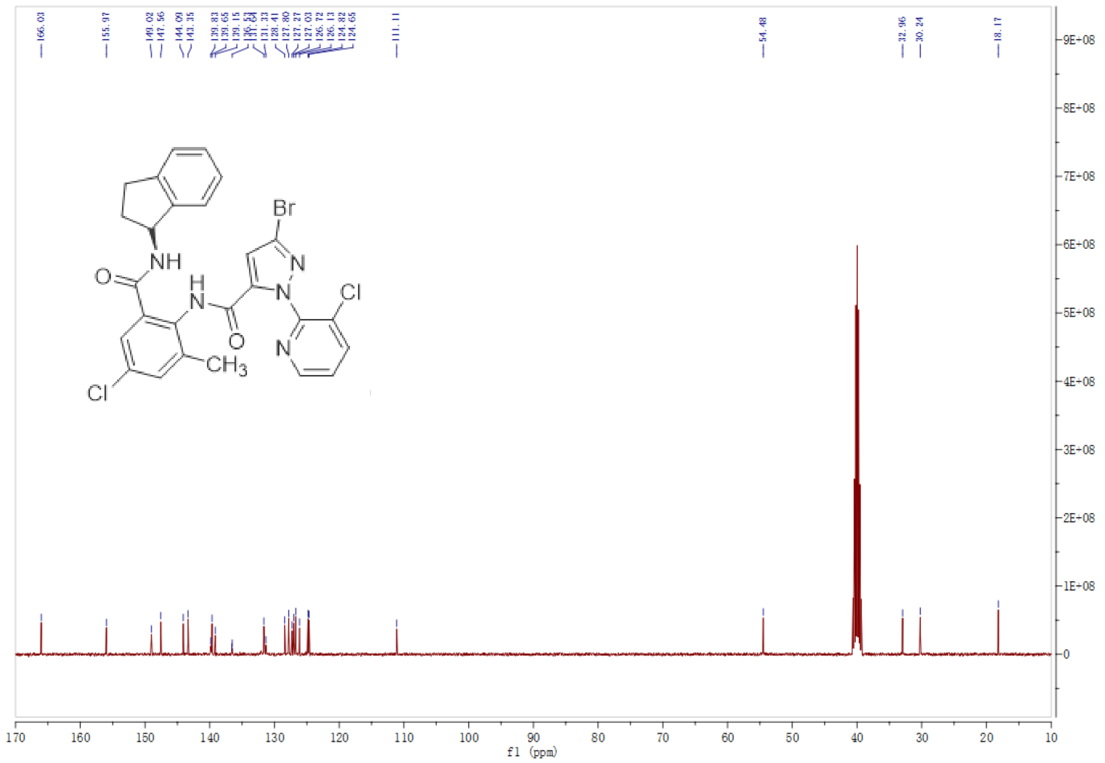

Figure S10. The ^{13}C NMR spectrum of **8e** (DMSO-*d*6).

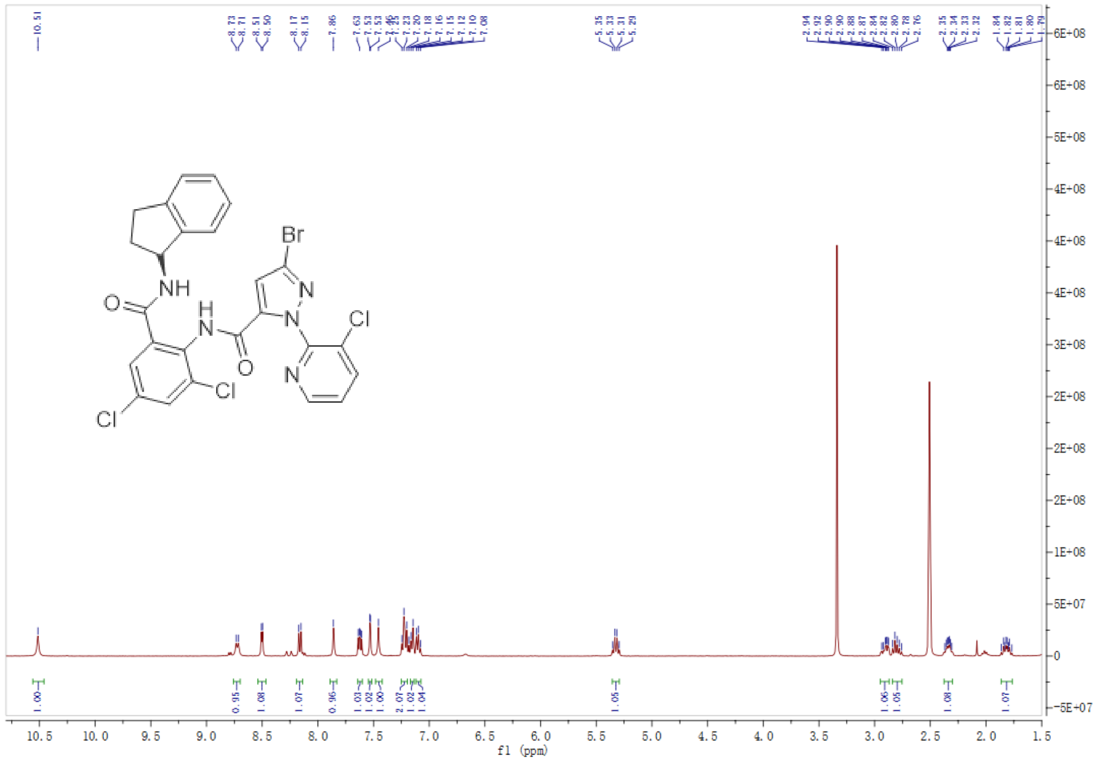

Figure S11. The ^{1}H NMR spectrum of **8f** (DMSO-d_6).

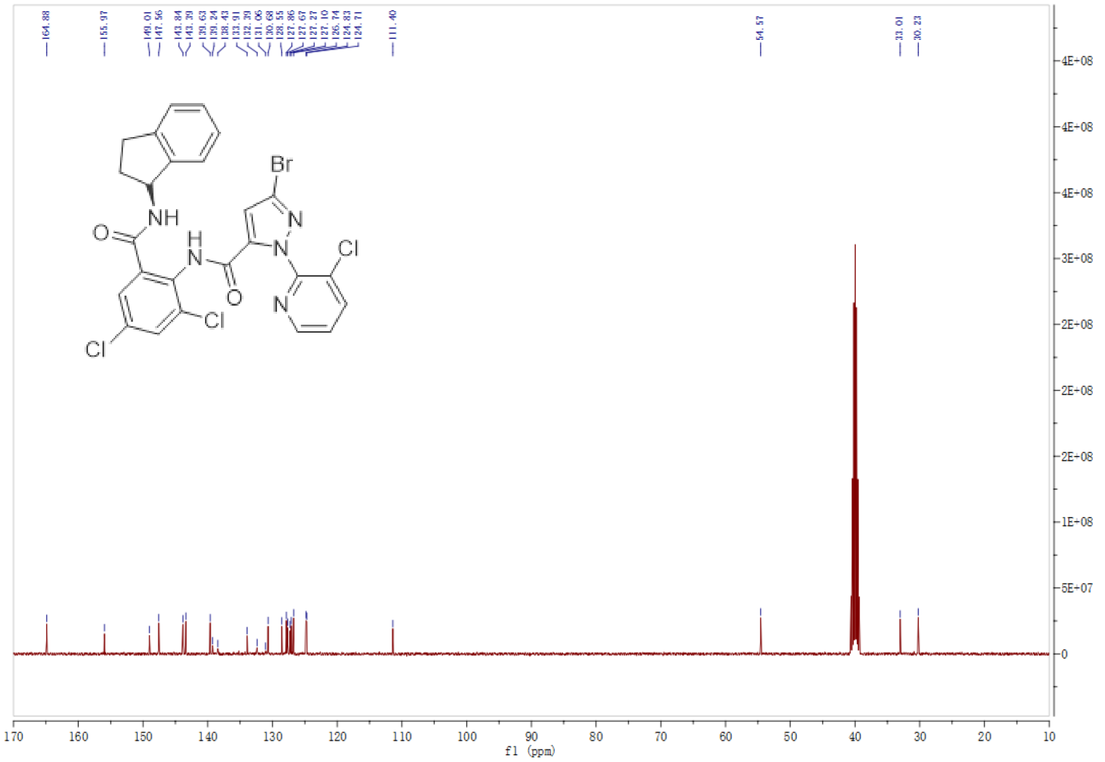

Figure S12. The ^{13}C NMR spectrum of **8f** (DMSO-d_6).

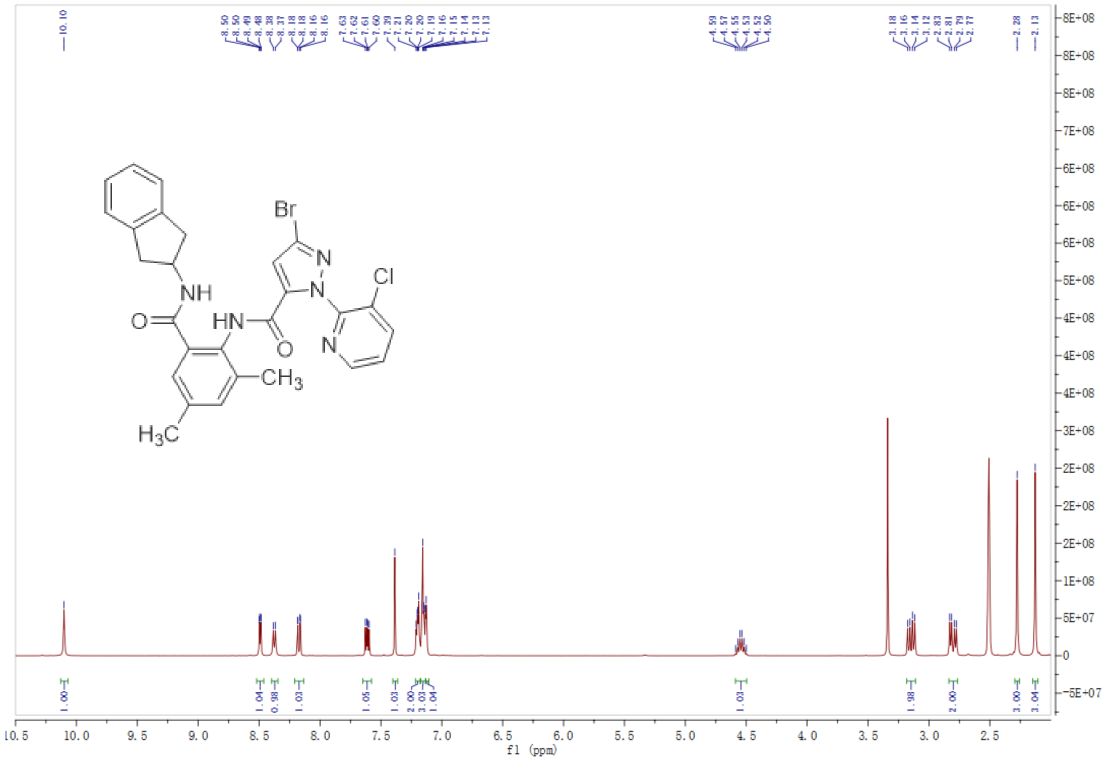

Figure S13. The ^{1}H NMR spectrum of **8g** (DMSO-d_6).

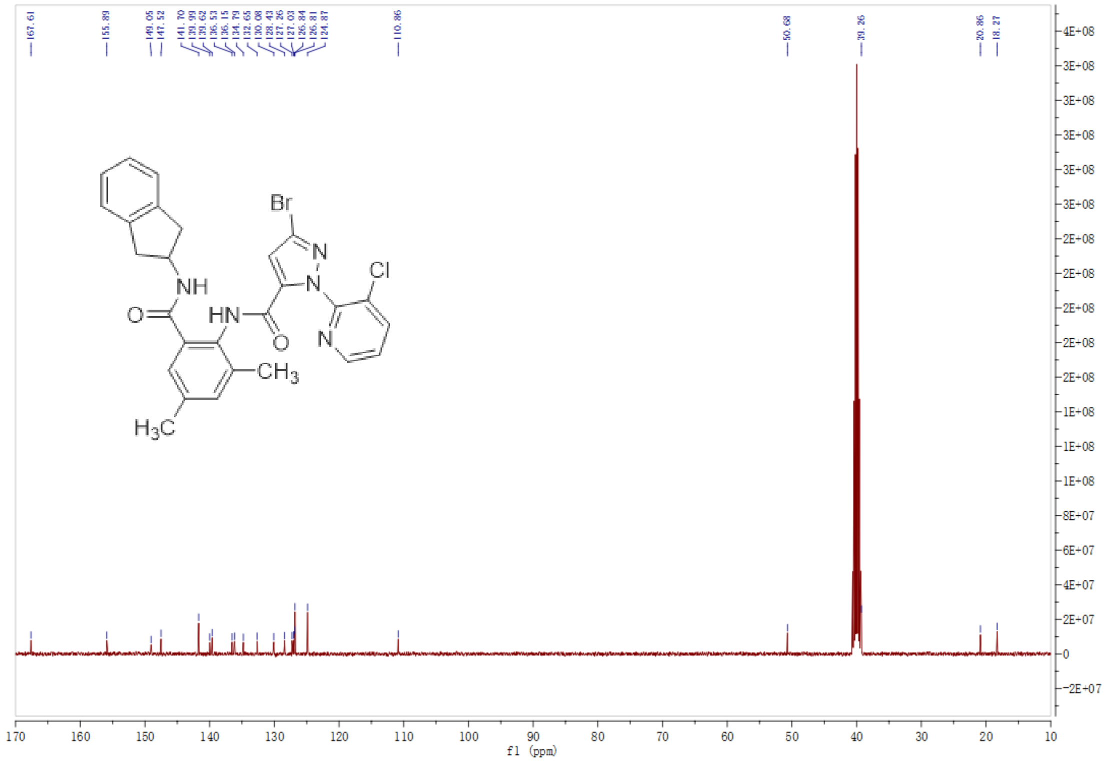

Figure S14. The ^{13}C NMR spectrum of **8g** (DMSO-d_6).

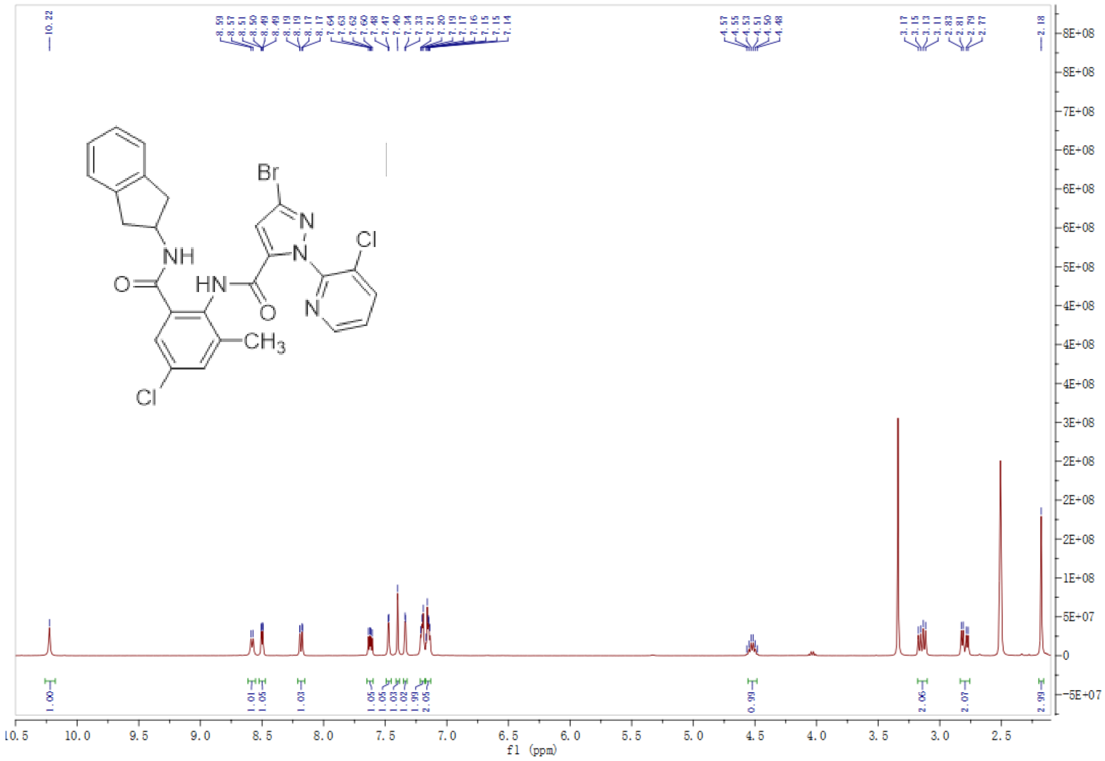

Figure S15. The ^{1}H NMR spectrum of **8h** (DMSO-d_6).

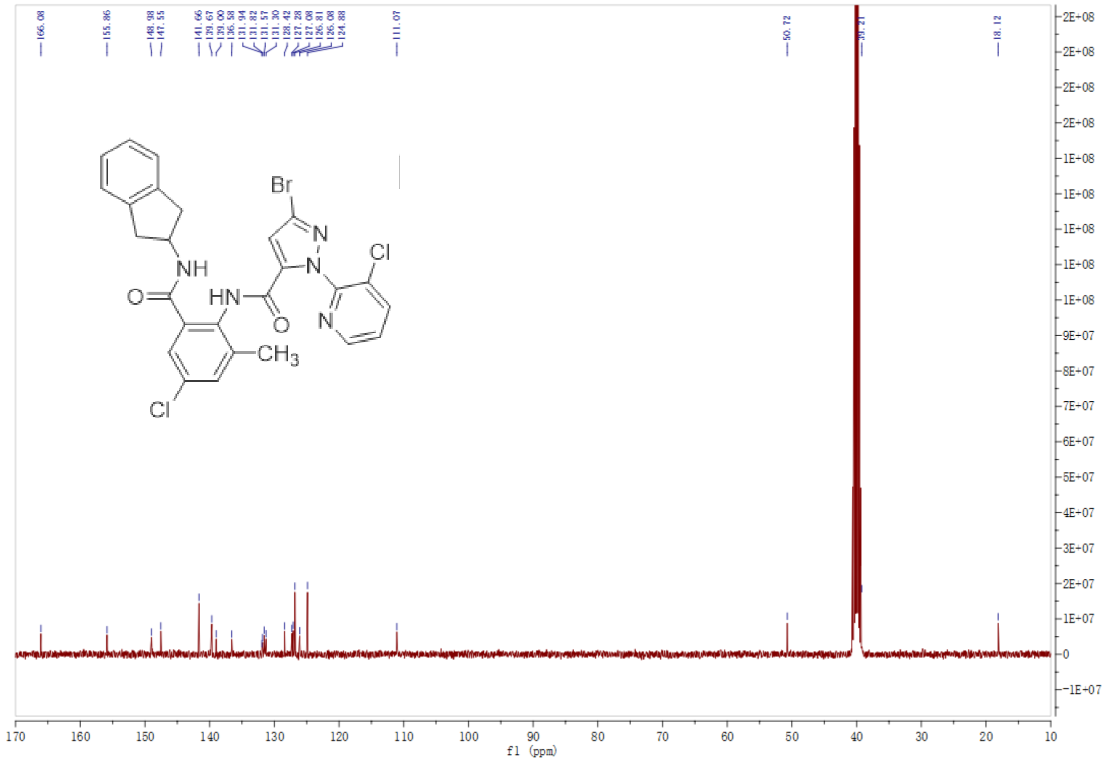

Figure S16. The ^{13}C NMR spectrum of **8h** (DMSO-d_6).

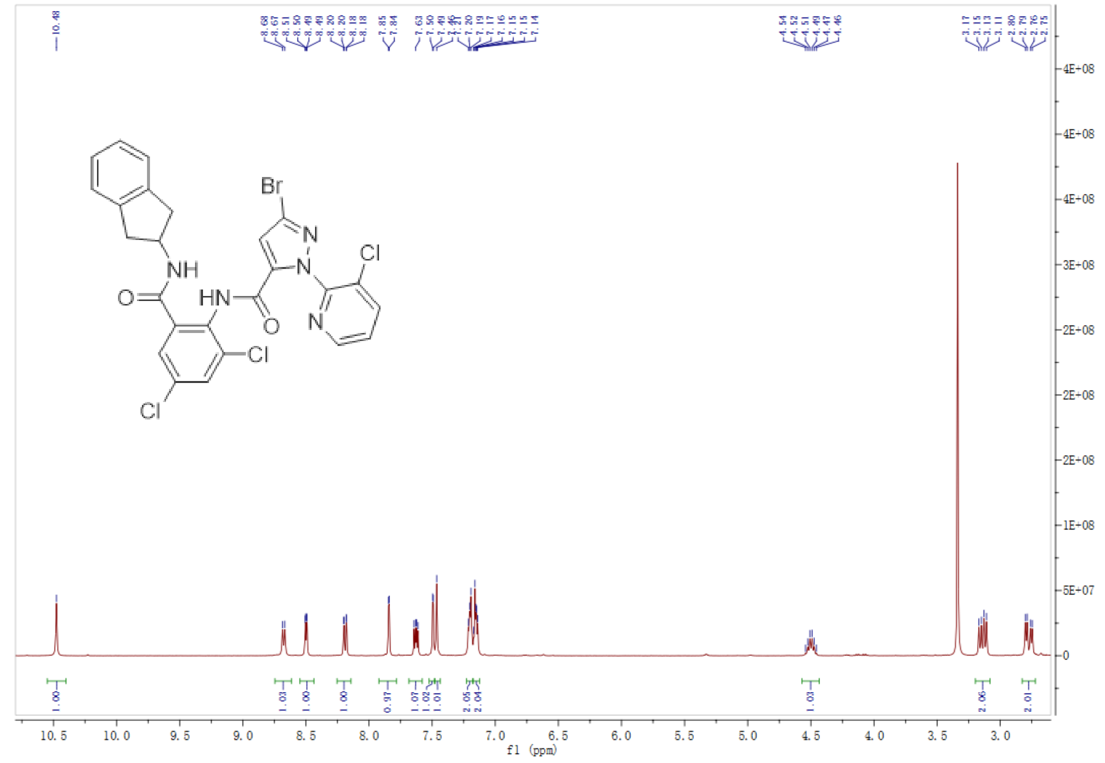

Figure S17. The ^{1}H NMR spectrum of **8i** (DMSO-d_6).

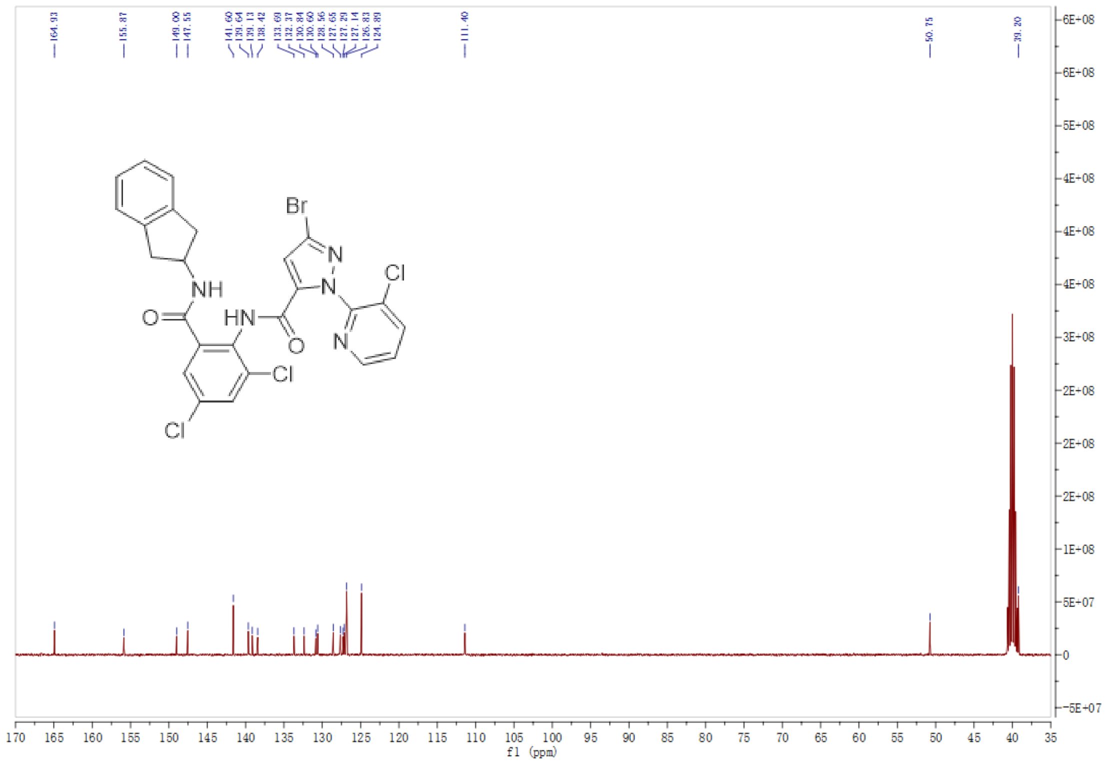

Figure S18. The ^{13}C NMR spectrum of **8i** (DMSO-d_6).

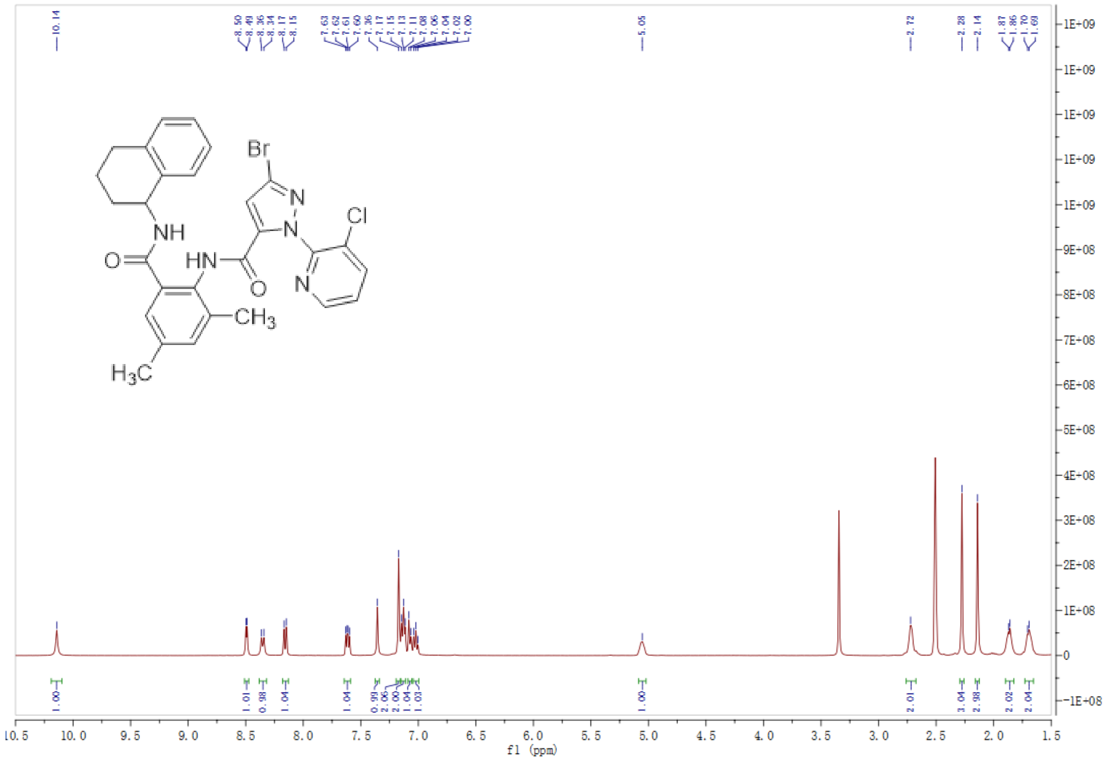

Figure S19. The ^{1}H NMR spectrum of **8j** (DMSO-d_6).

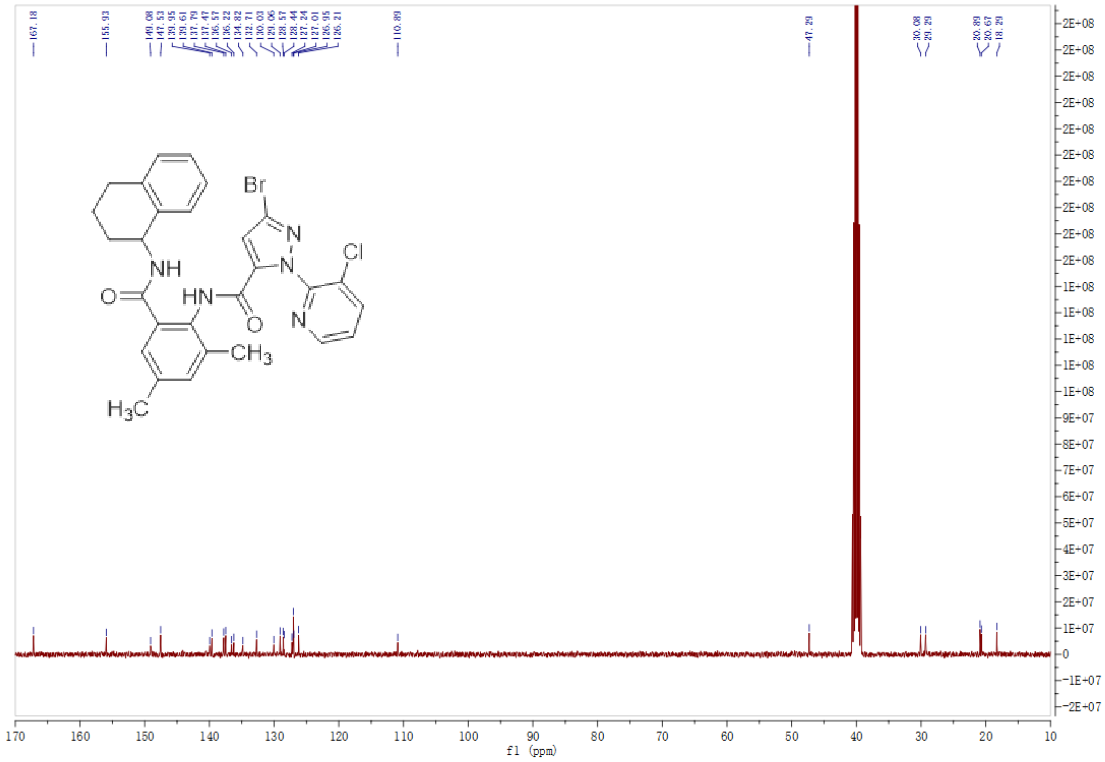

Figure S20. The ^{13}C NMR spectrum of **8j** (DMSO-d_6).

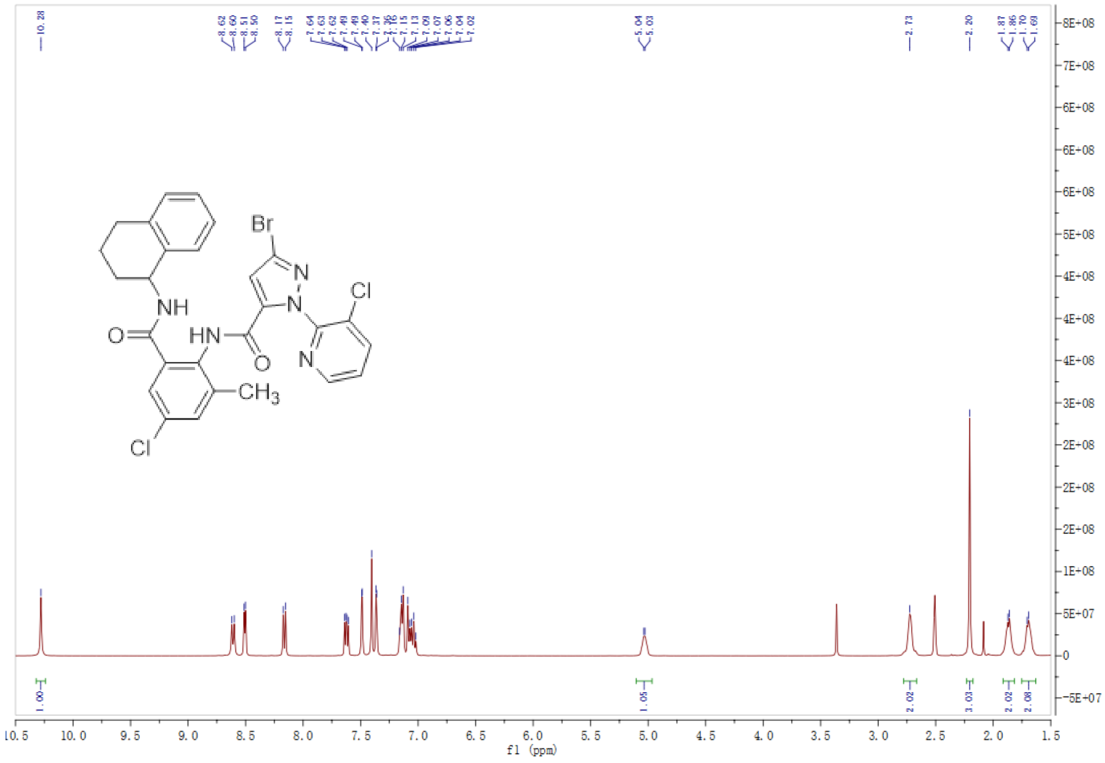

Figure S21. The ^{1}H NMR spectrum of **8k** (DMSO-d_6).

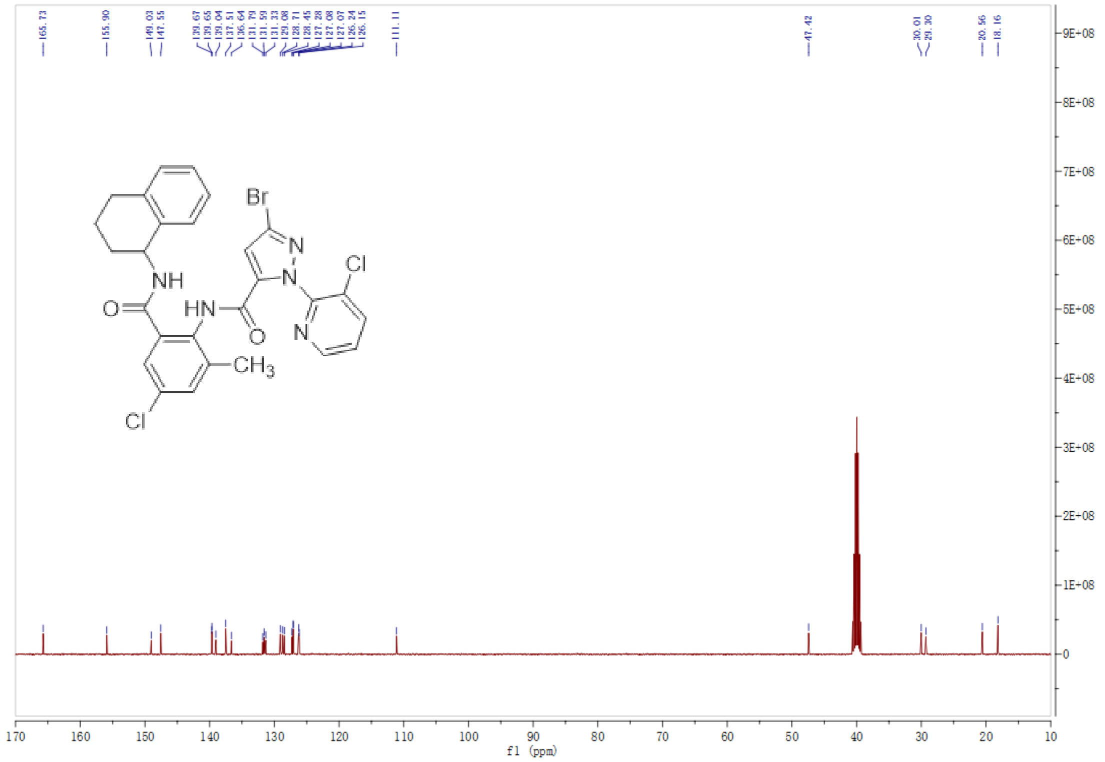

Figure S22. The ^{13}C NMR spectrum of **8k** (DMSO-d_6).

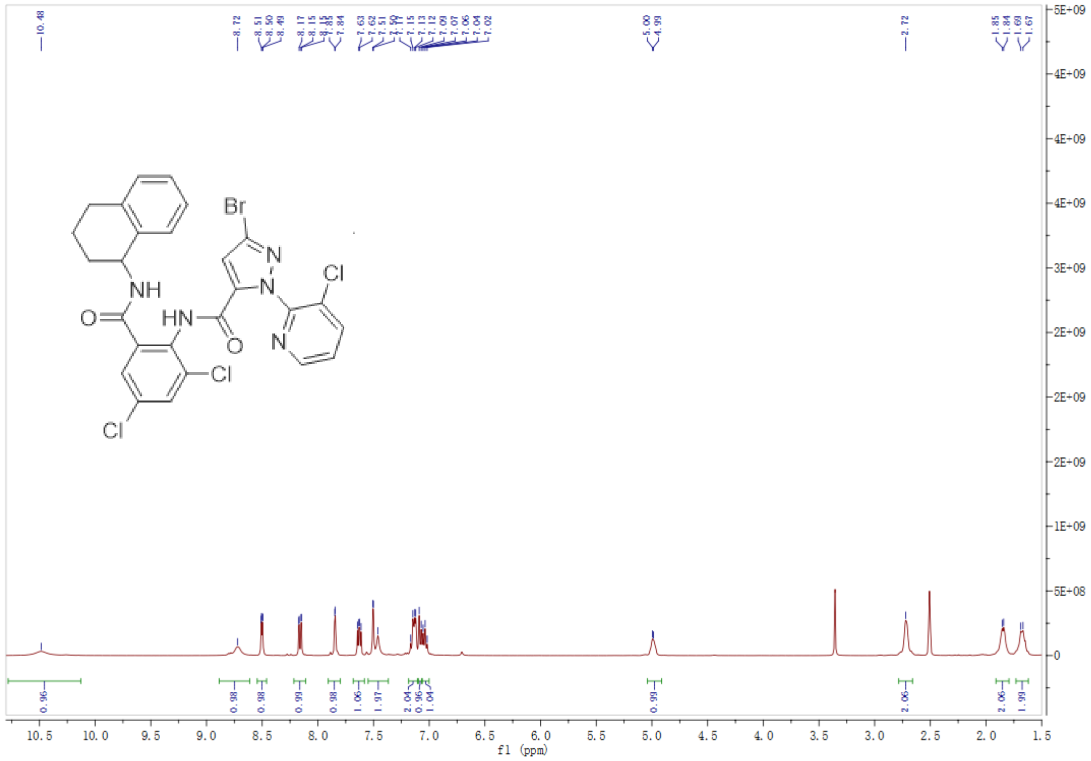

Figure S23. The ^{1}H NMR spectrum of **8l** (DMSO-d_6).

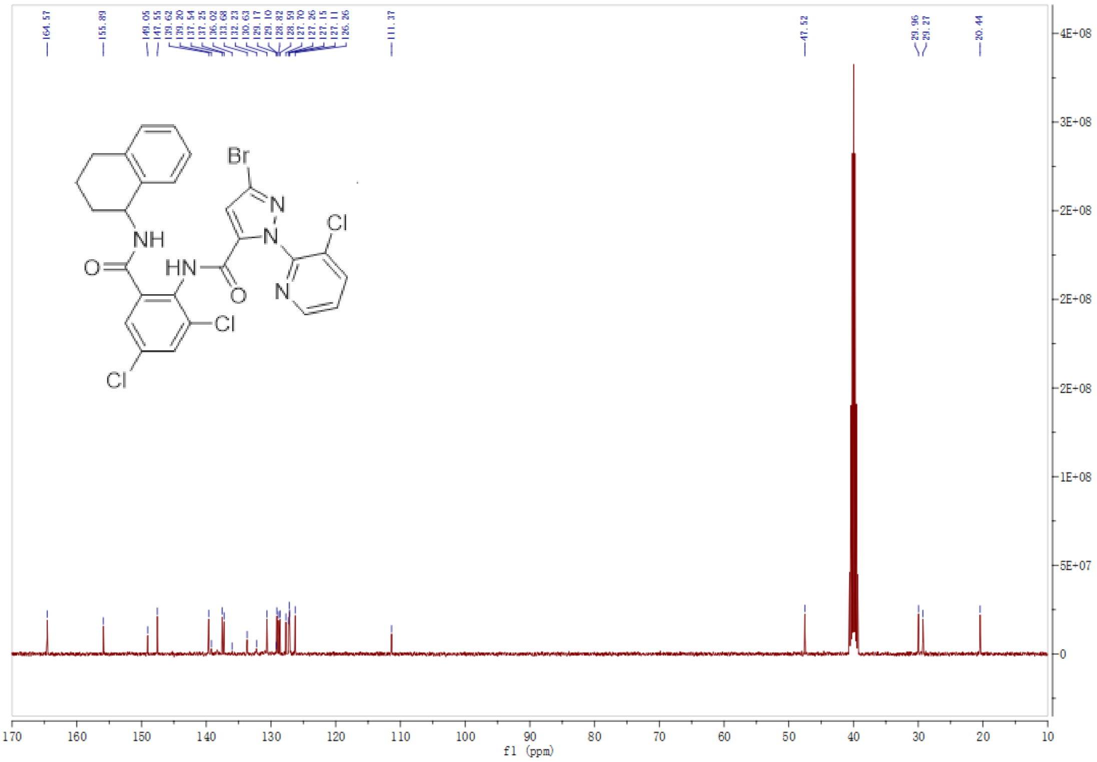

Figure S24. The ^{13}C NMR spectrum of **8l** (DMSO-d_6).

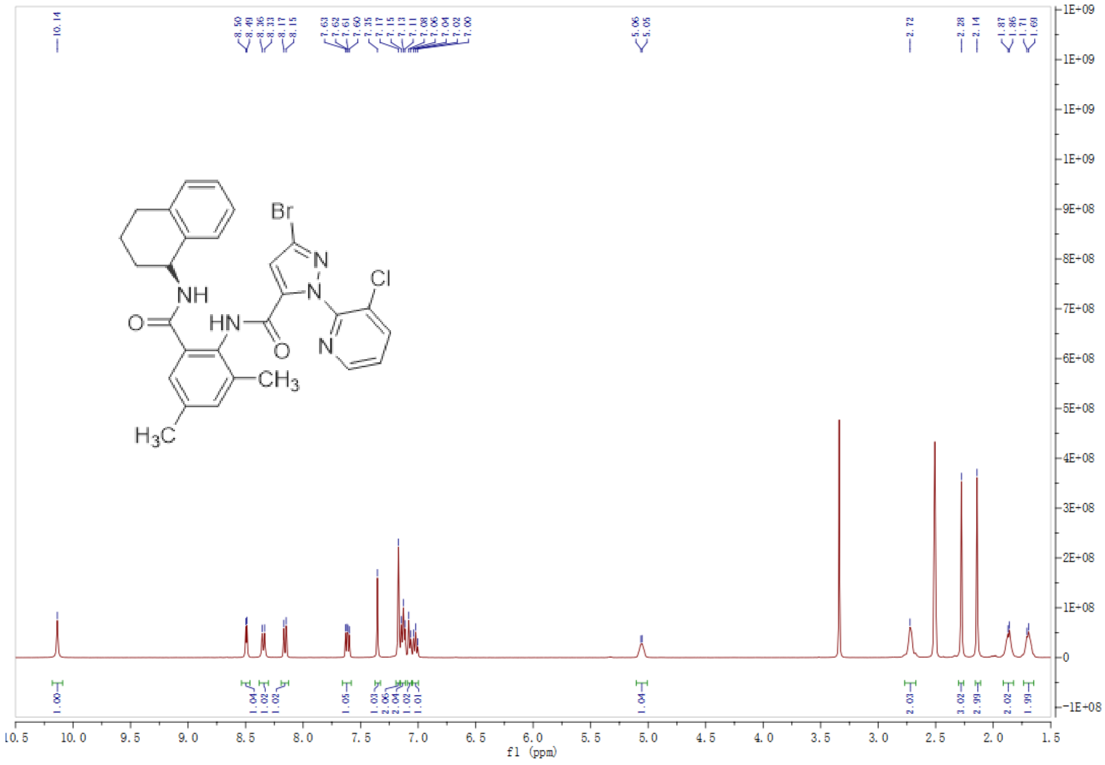

Figure S25. The ^{1}H NMR spectrum of **8m** (DMSO-d_6).

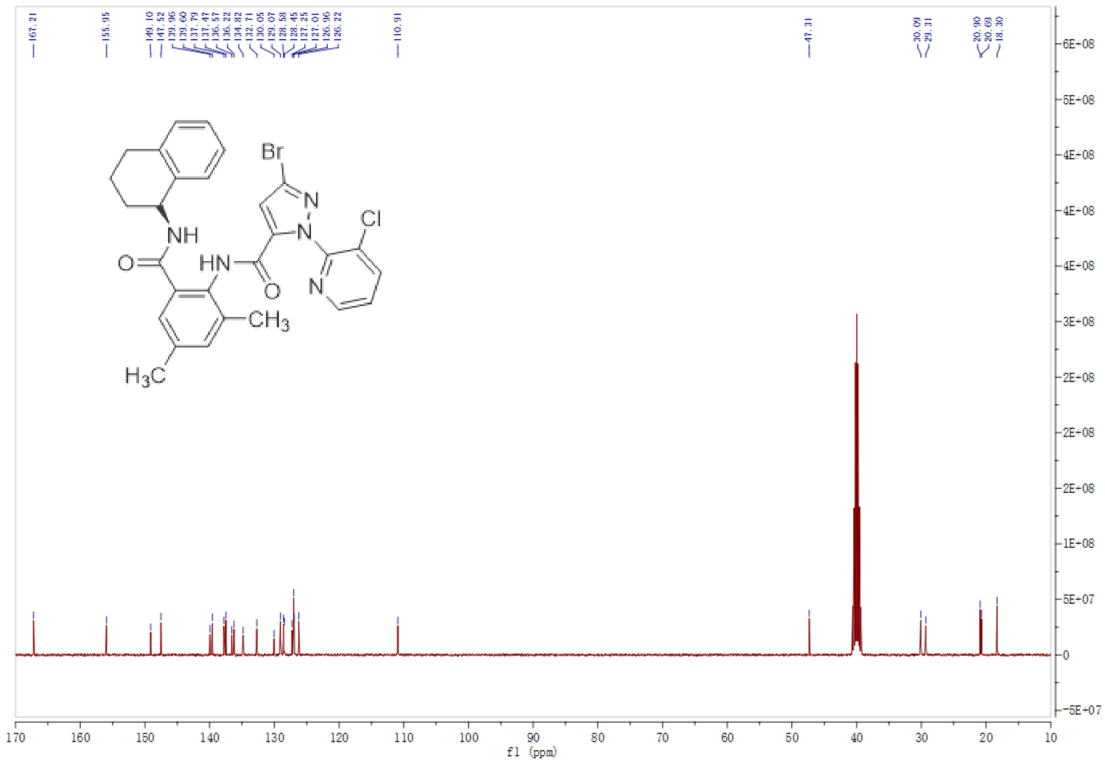

Figure S26. The ^{13}C NMR spectrum of **8m** (DMSO-d_6).

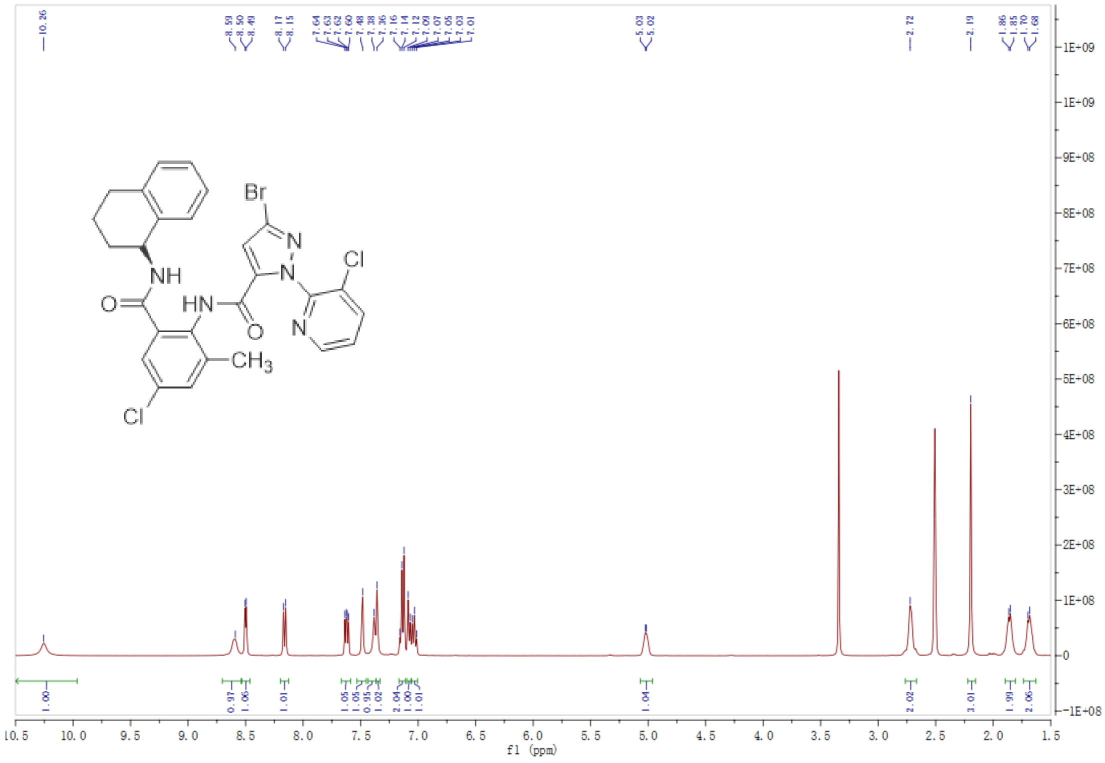

Figure S27. The ^{1}H NMR spectrum of **8n** (DMSO-d_6).

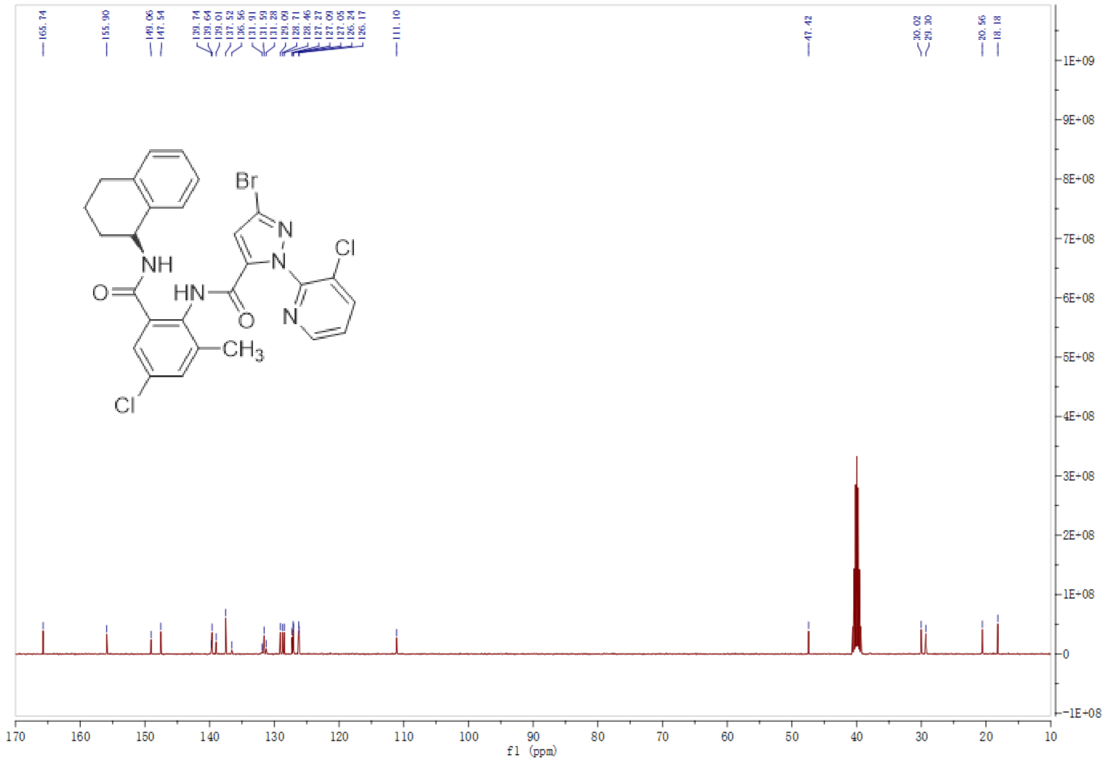

Figure S28. The ^{13}C NMR spectrum of **8n** (DMSO-d_6).

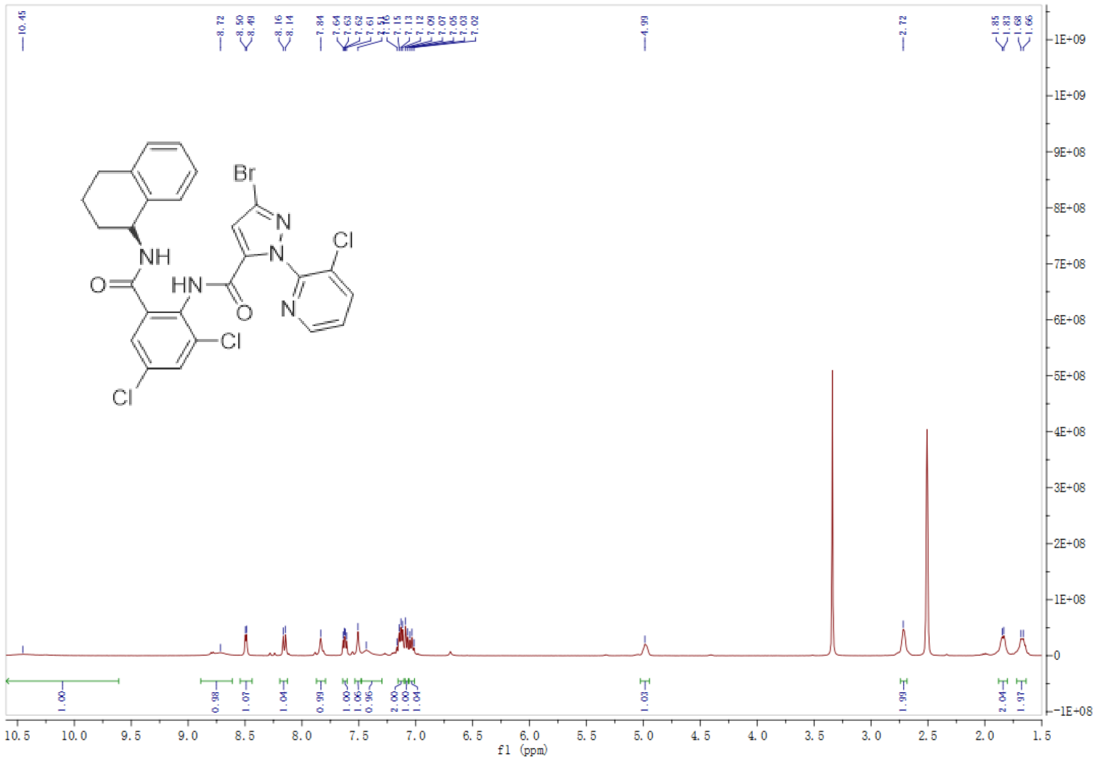

Figure S29. The ^{1}H NMR spectrum of **8o** (DMSO-*d*$_6$).

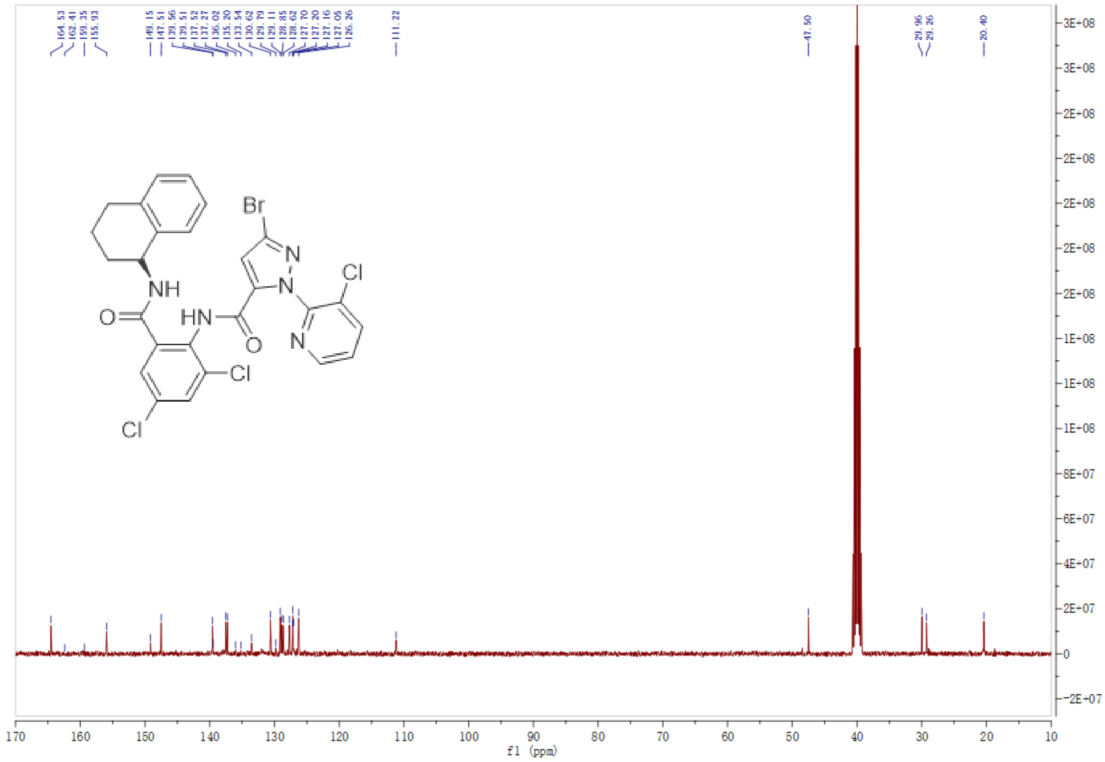

Figure S30. The ^{13}C NMR spectrum of **8o** (DMSO-*d*$_6$).

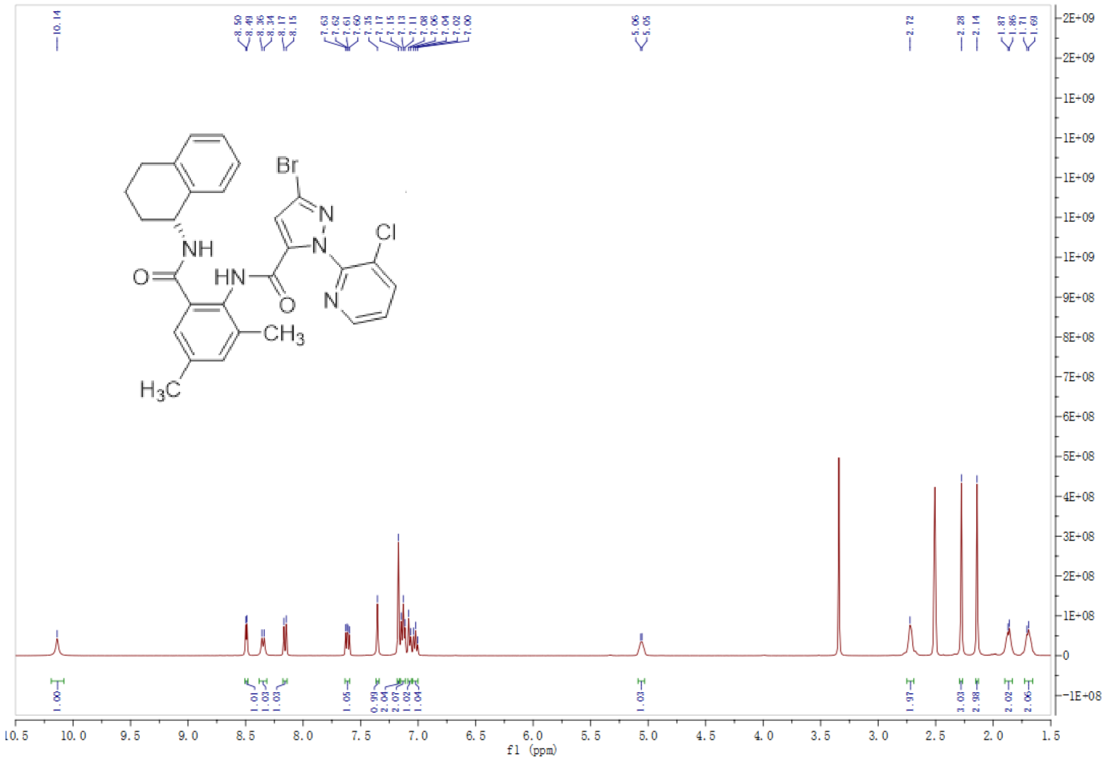

Figure S31. The ^{1}H NMR spectrum of **8p** (DMSO-d_6).

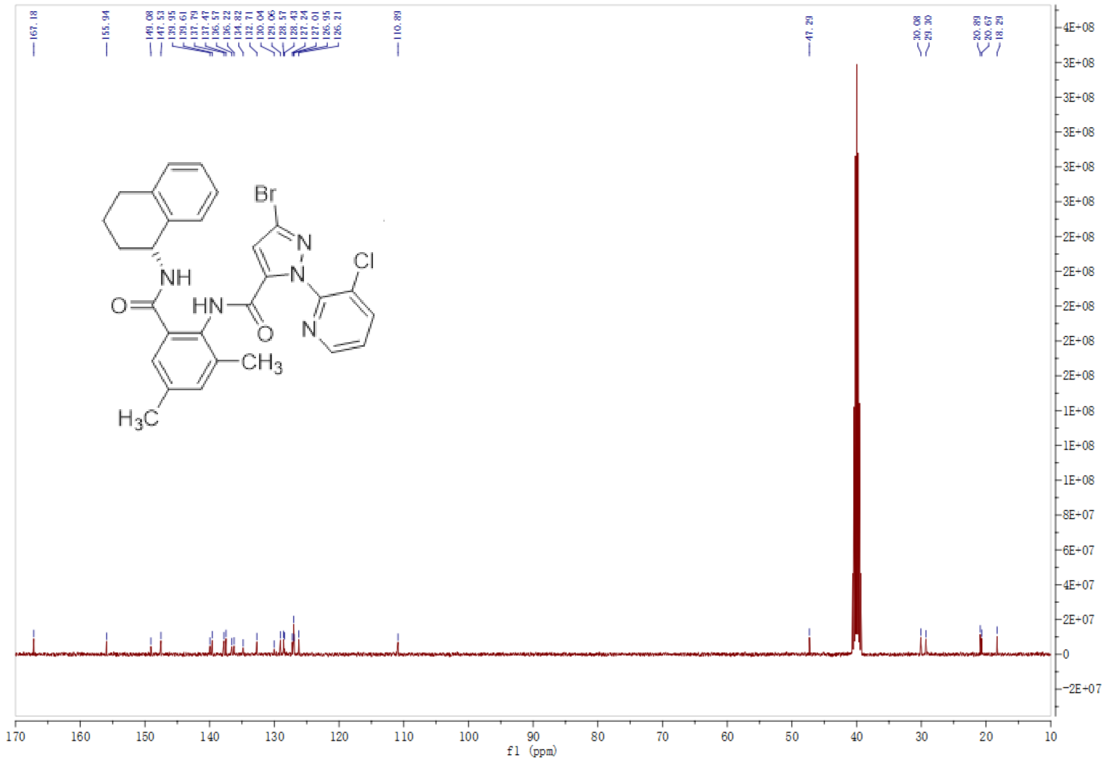

Figure S32. The ^{13}C NMR spectrum of **8p** (DMSO-d_6).

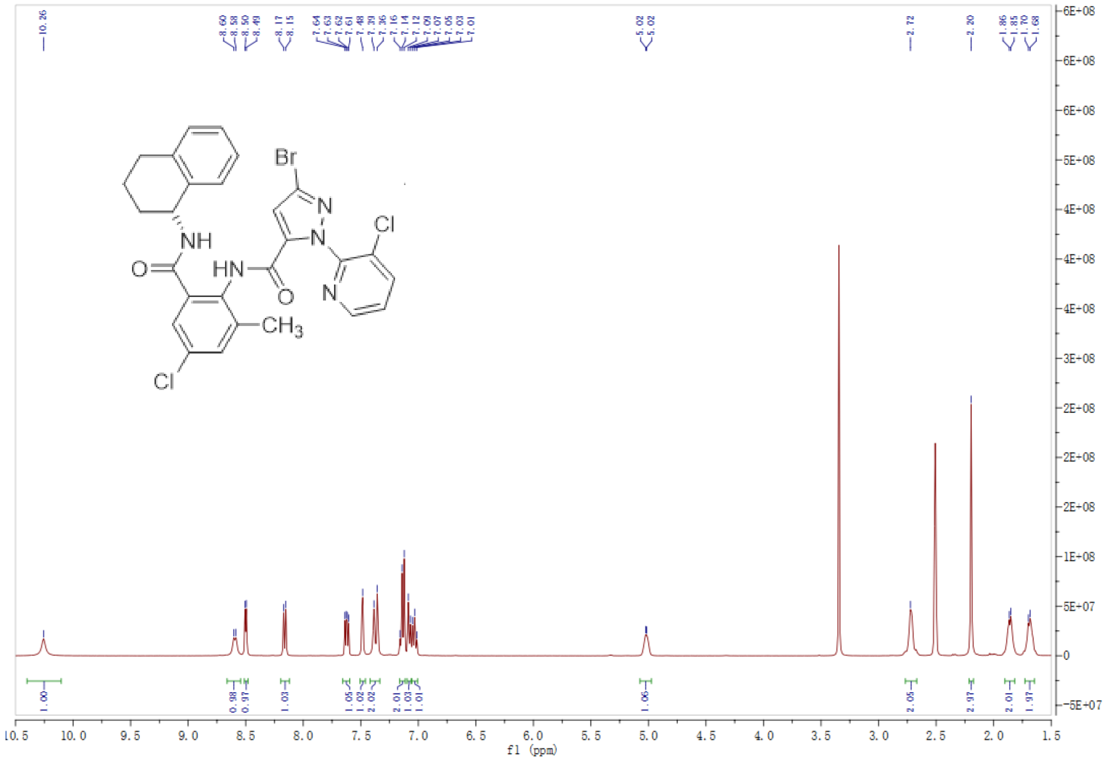

Figure S33. The ^{1}H NMR spectrum of **8q** (DMSO-*d*$_6$).

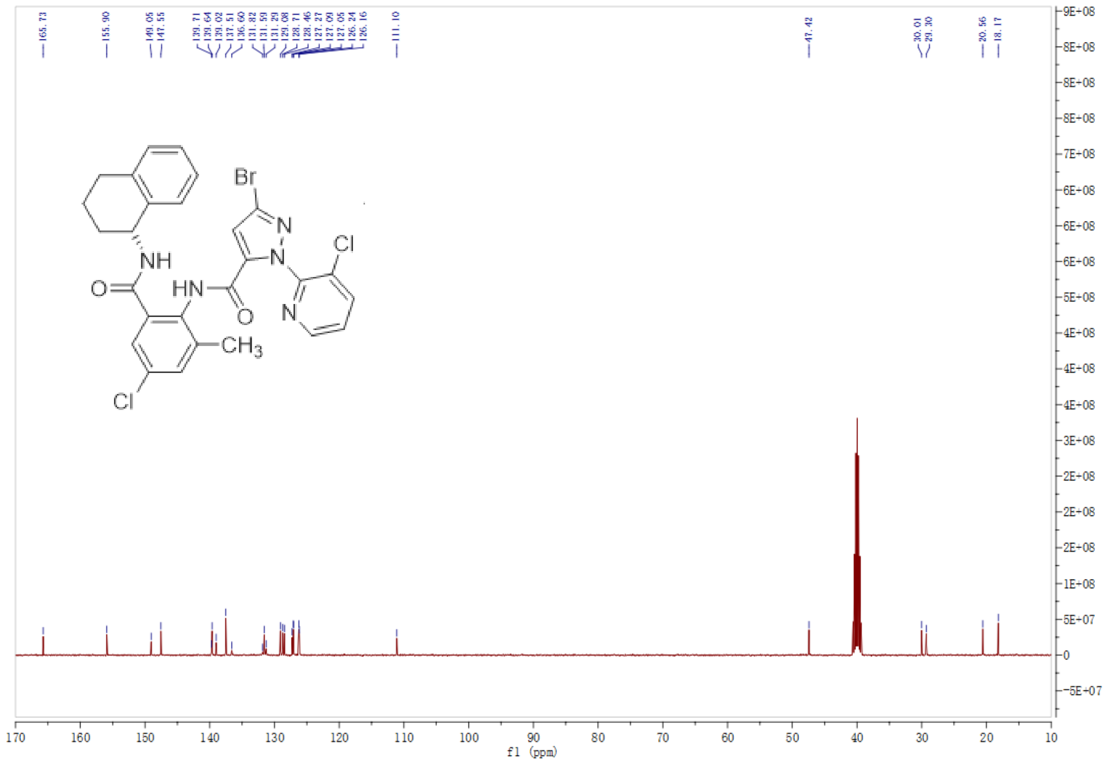

Figure S34. The ^{13}C NMR spectrum of **8q** (DMSO-*d*$_6$).

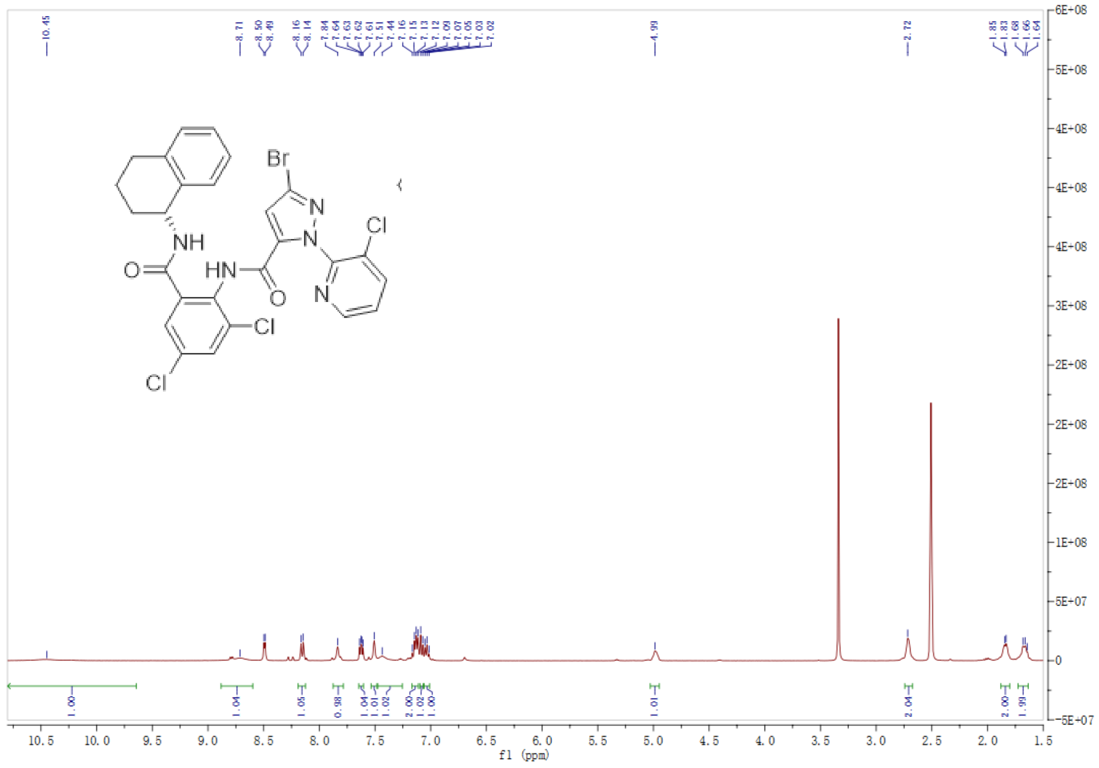

Figure S35. The ¹H NMR spectrum of **8r** (DMSO-d_6).

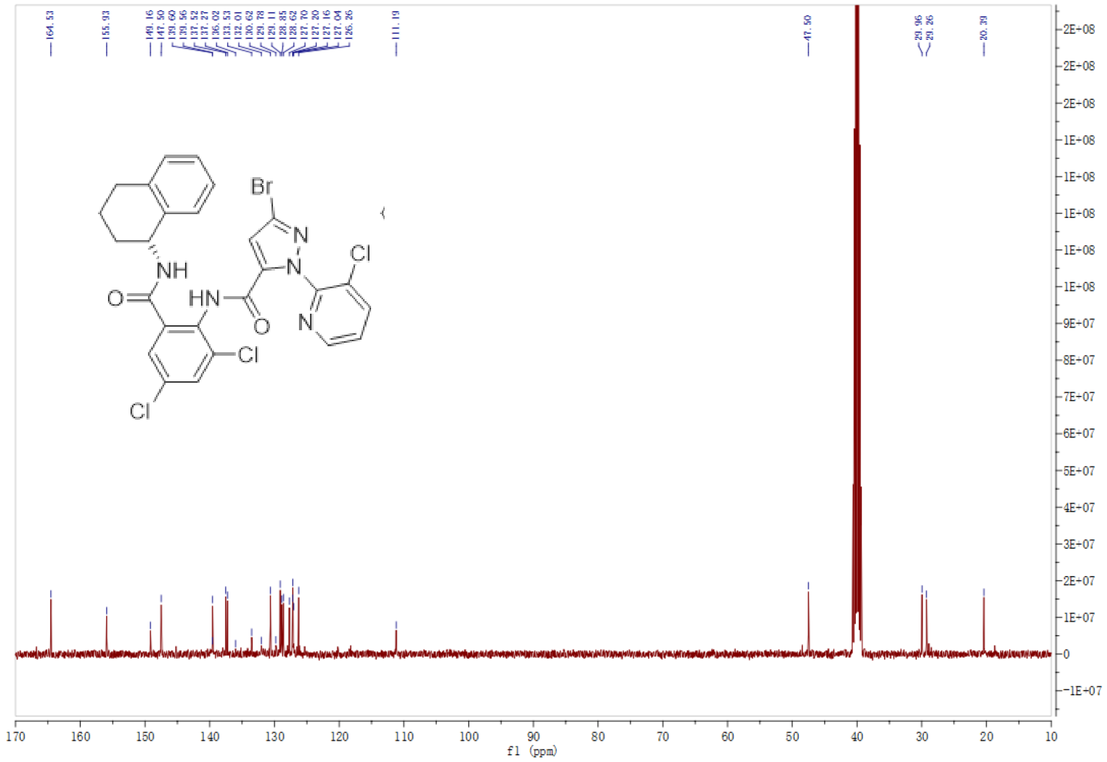

Figure S36. The ¹³C NMR spectrum of **8r** (DMSO-d_6).

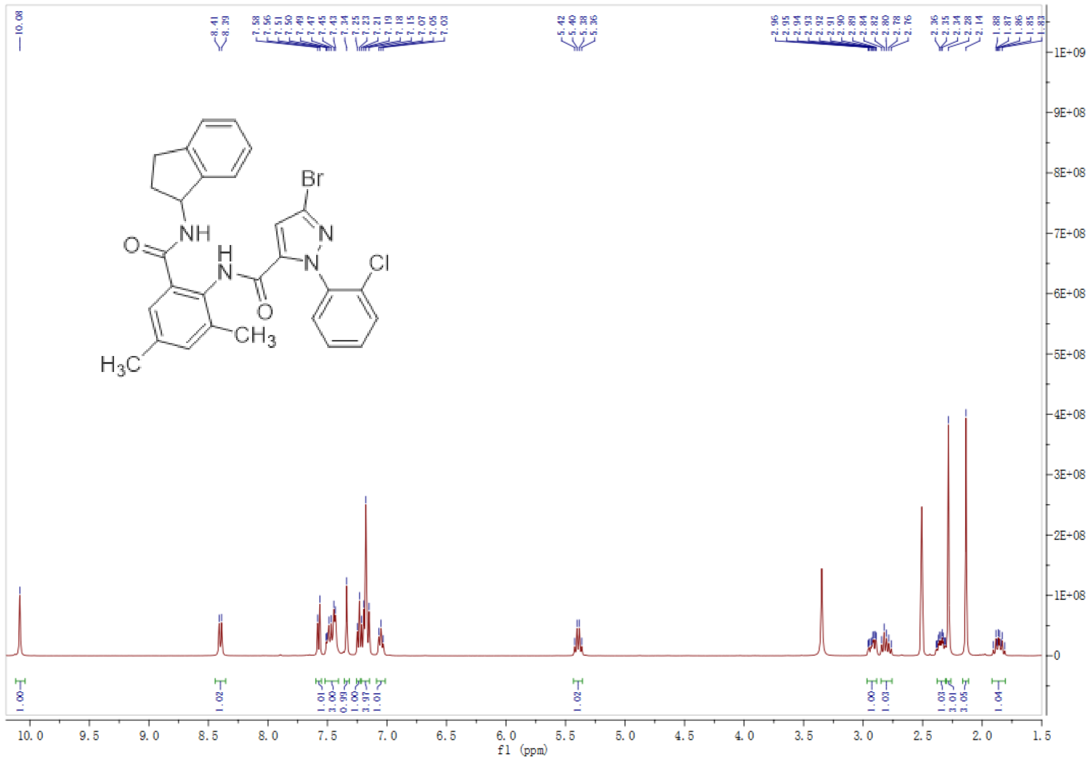

Figure S37. The ^{1}H NMR spectrum of **8s** (DMSO-d_6).

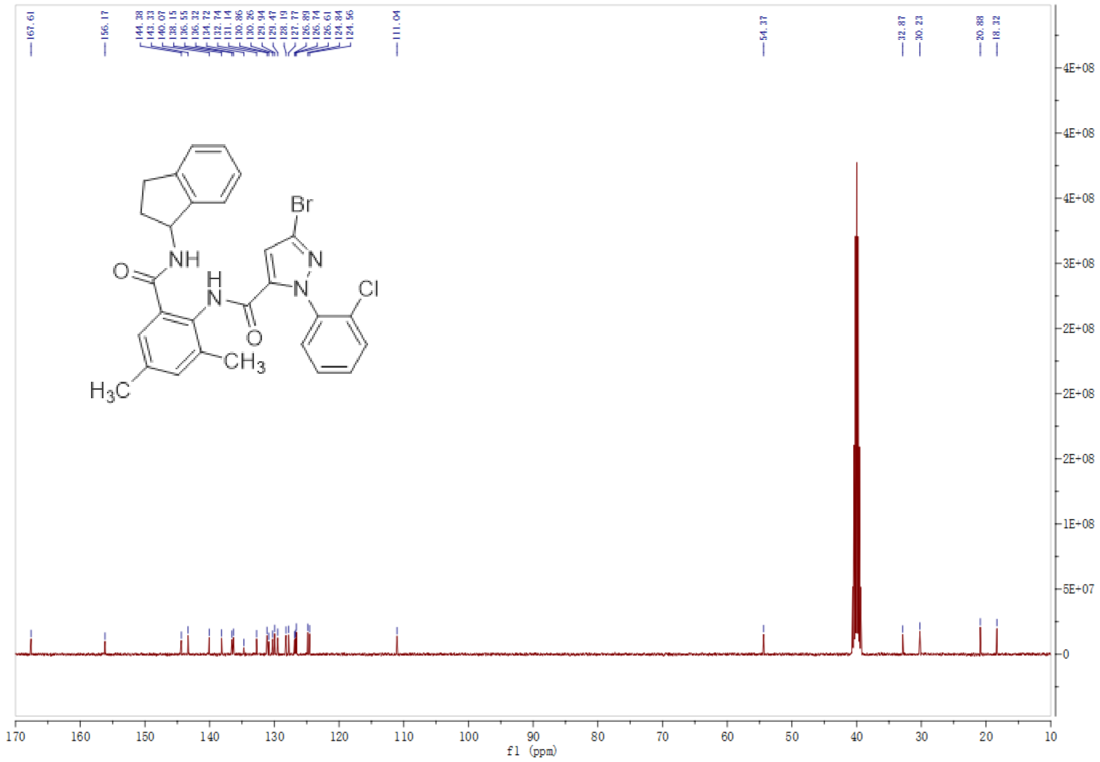

Figure S38. The ^{13}C NMR spectrum of **8s** (DMSO-d_6).

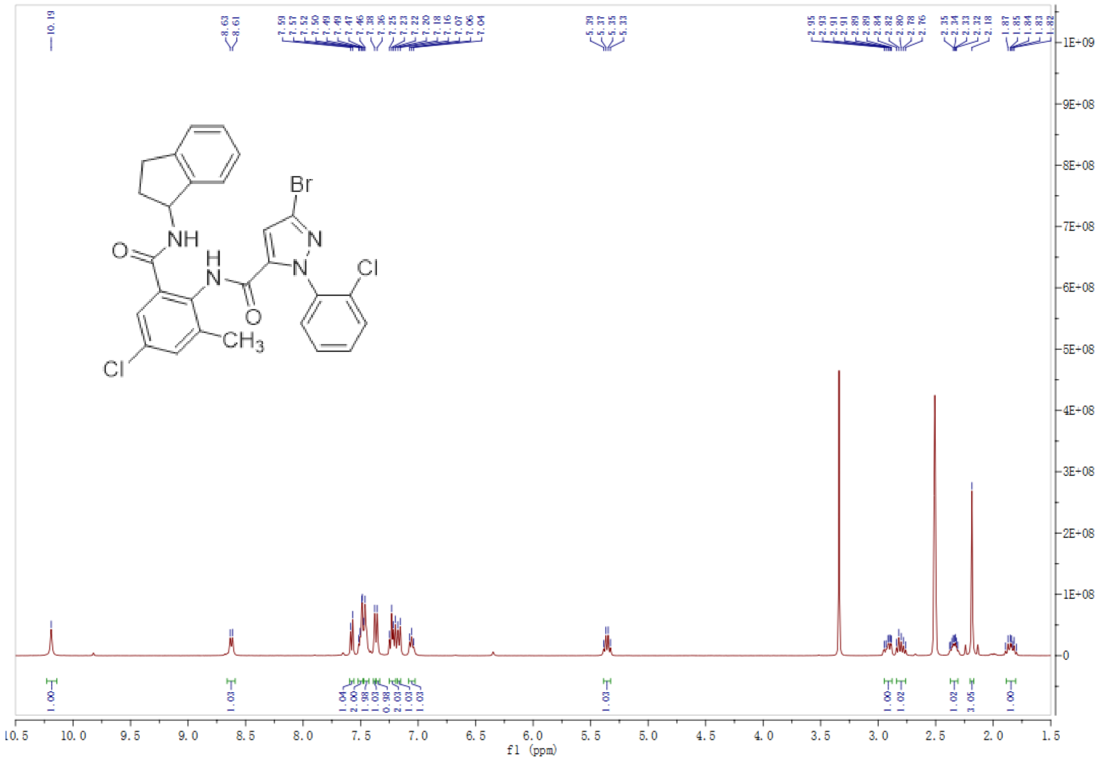

Figure S39. The ¹H NMR spectrum of **8t** (DMSO-d_6).

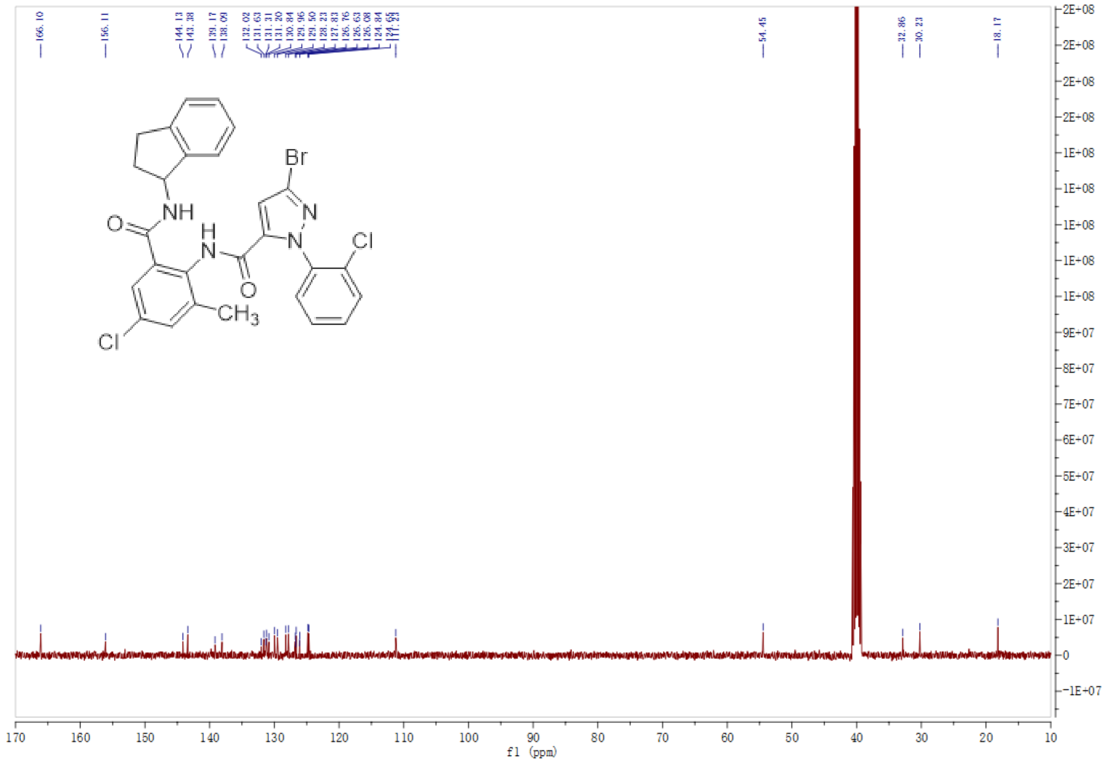

Figure S40. The ¹³C NMR spectrum of **8t** (DMSO-d_6).

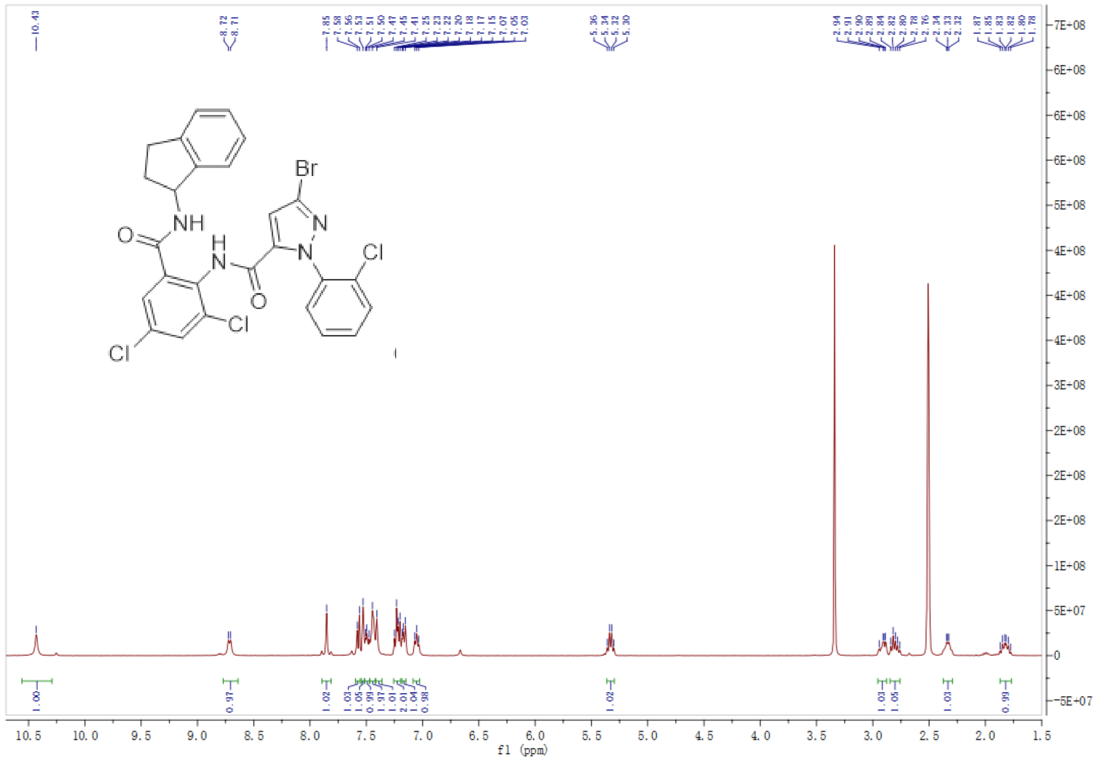

Figure S41. The ^{1}H NMR spectrum of **8u** (DMSO-d_6).

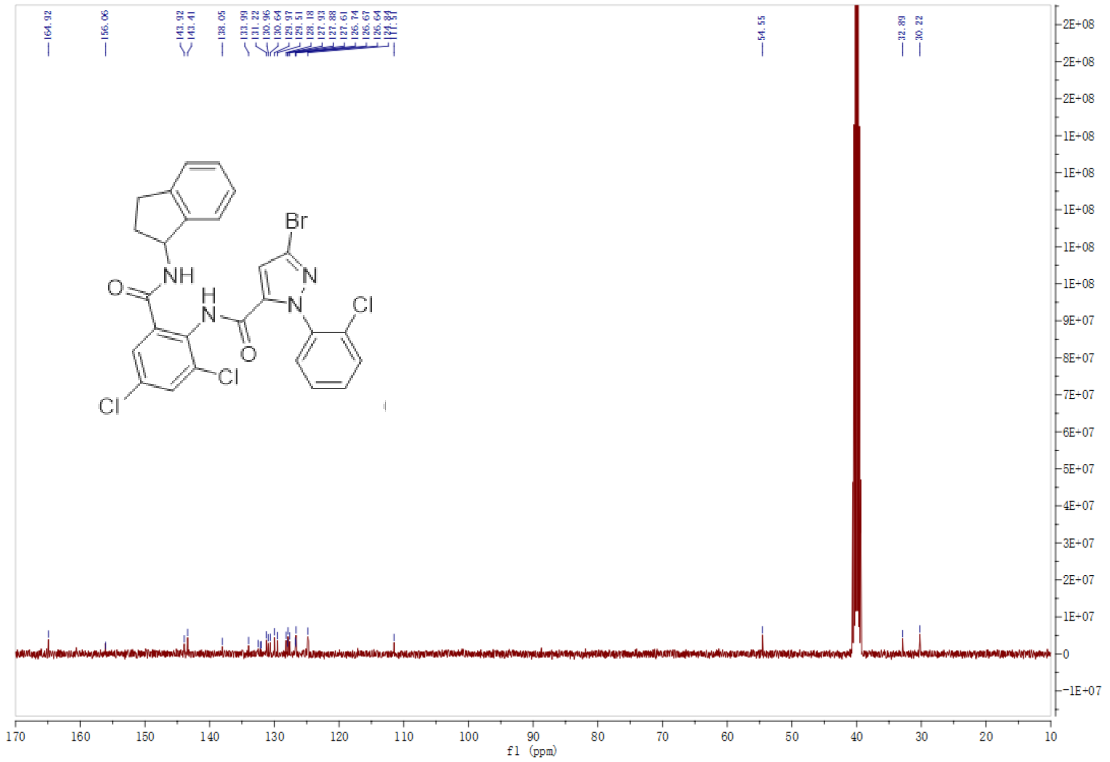

Figure S42. The ^{13}C NMR spectrum of **8u** (DMSO-d_6).

2. Safety assessment data of compound 8q and chlorantraniliprole.

CNC(=O)c1cc(Cl)cc(C)c1NC(=O)c1cc(Br)nn1-c1ncccc1Cl

Download as CSV | Download as PDF

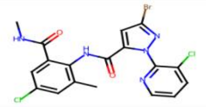

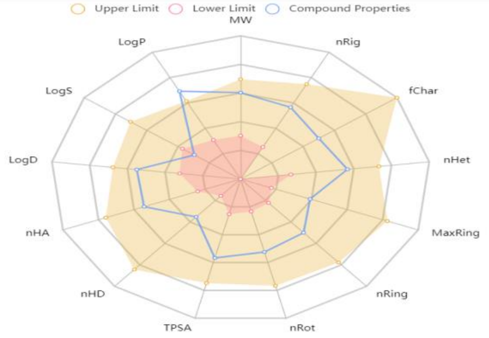

Physicochemical Property	
Molecular Weight (MW)	480.970
Volume	390.120
Density	1.233
nHA	7
nHD	2
nRot	6
nRing	3
MaxRing	6
nHet	10
fChar	0
nRig	19
Flexibility	0.316
Stereo Centers	0
TPSA	88.910
logS	-5.034
logP	3.761
logD	2.284

Medicinal Chemistry	
QED	0.581
SAscore	2.578
Fsp^3	0.111
MCE-18	20.000
NPscore	-1.726
Lipinski Rule	Accepted
Pfizer Rule	Accepted
GSK Rule	Rejected
Golden Triangle	Accepted
PAINS	0 alert(s)
ALARM NMR Rule	1 alert(s) DETAIL
BMS Rule	0 alert(s)
Chelator Rule	0 alert(s)
**Absorption**	
Caco-2 Permeability	-4.804
MDCK Permeability	1.6e-05
Pgp-inhibitor	--
Pgp-substrate	---
HIA	---
$F_{20\%}$	---
$F_{30\%}$	---
**Distribution**	
PPB	97.485%
VD	0.758
BBB Penetration	+++
Fu	1.508%
**Metabolism**	
CYP1A2 inhibitor	+
CYP1A2 substrate	-
CYP2C19 inhibitor	++
CYP2C19 substrate	+
CYP2C9 inhibitor	+++
CYP2C9 substrate	++
CYP2D6 inhibitor	---
CYP2D6 substrate	-
CYP3A4 inhibitor	+++
CYP3A4 substrate	-
**Excretion**	
CL	2.080
$T_{1/2}$	0.207

Toxicity	
hERG Blockers	---
H-HT	-
DILI	+++
AMES Toxicity	---
Rat Oral Acute Toxicity	+
FDAMDD	++
Skin Sensitization	---
Carcinogencity	-
Eye Corrosion	---
Eye Irritation	---
Respiratory Toxicity	---
**Environmental Toxicity**	
Bioconcentration Factors	1.081
IGC_{50}	4.284
$LC_{50}FM$	5.602
$LC_{50}DM$	5.187
**Tox21 Pathway**	
NR-AR	---
NR-AR-LBD	---
NR-AhR	+++
NR-Aromatase	--
NR-ER	--
NR-ER-LBD	---
NR-PPAR-gamma	-
SR-ARE	+
SR-ATAD5	+
SR-HSE	---
SR-MMP	++
SR-p53	++
**Toxicophore Rules**	
Acute Toxicity Rule	0 alert(s)
Genotoxic Carcinogenicity Rule	0 alert(s)
NonGenotoxic Carcinogenicity Rule	1 alert(s) DETAIL
Skin Sensitization Rule	4 alert(s) DETAIL
Aquatic Toxicity Rule	1 alert(s) DETAIL
NonBiodegradable Rule	2 alert(s) DETAIL
SureChEMBL Rule	0 alert(s)
FAF-Drugs4 Rule	1 alert(s) DETAIL

Tip: For the classification endpoints , the prediction probability values are transformed into six symbols: 0-0.1(---), 0.1-0.3(--), 0.3-0.5(-), 0.5-0.7(+), 0.7-0.9(++), and 0.9-1.0(+++).

Figure S43. The safety assessment data of chlorantraniliprole.

Cc1cc(Cl)cc(C(=O)N[C@@H]2CCCc3ccccc32)c1NC(=O)c1cc(Br)nn1-c1ncccc1Cl

Download as CSV
Download as PDF

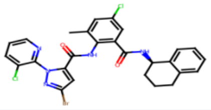

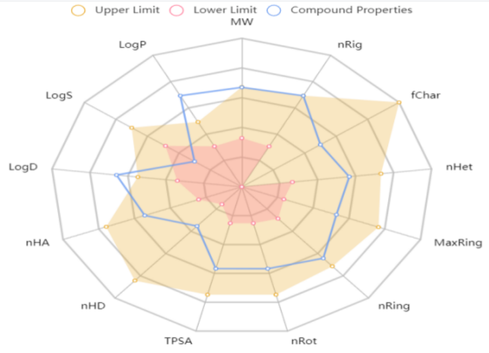

Physicochemical Property

Molecular Weight (MW)	597.030
Volume	520.762
Density	1.146
nHA	7
nHD	2
nRot	7
nRing	5
MaxRing	10
nHet	10
fChar	0
nRig	30
Flexibility	0.233
Stereo Centers	1
TPSA	88.910
logS	-7.868
logP	6.222
logD	4.081

Medicinal Chemistry

QED	0.267
SAscore	3.152
Fsp^3	0.185
MCE-18	94.250
NPscore	-1.443
Lipinski Rule	Rejected
Pfizer Rule	Accepted
GSK Rule	Rejected
Golden Triangle	Rejected
PAINS	0 alert(s)
ALARM NMR Rule	1 alert(s) DETAIL
BMS Rule	0 alert(s)
Chelator Rule	0 alert(s)

Absorption

Caco-2 Permeability	-5.092
MDCK Permeability	9.3e-06
Pgp-inhibitor	+++
Pgp-substrate	---
HIA	---
$F_{20\%}$	---
$F_{30\%}$	---

Distribution

PPB	99.245%
VD	0.780
BBB Penetration	+++
Fu	1.523%

Metabolism

CYP1A2 inhibitor	--
CYP1A2 substrate	-
CYP2C19 inhibitor	+++
CYP2C19 substrate	-
CYP2C9 inhibitor	+++
CYP2C9 substrate	+++
CYP2D6 inhibitor	+
CYP2D6 substrate	++
CYP3A4 inhibitor	+++
CYP3A4 substrate	++

Excretion

CL	1.266
$T_{1/2}$	0.047

Toxicity

hERG Blockers	--
H-HT	++
DILI	+++
AMES Toxicity	-
Rat Oral Acute Toxicity	++
FDAMDD	+++
Skin Sensitization	---
Carcinogencity	--
Eye Corrosion	---
Eye Irritation	---
Respiratory Toxicity	---
**Environmental Toxicity**	
Bioconcentration Factors	1.769
IGC_{50}	5.068
$LC_{50}FM$	6.393
$LC_{50}DM$	5.698
**Tox21 Pathway**	
NR-AR	-
NR-AR-LBD	---
NR-AhR	+++
NR-Aromatase	++
NR-ER	+
NR-ER-LBD	---
NR-PPAR-gamma	+++
SR-ARE	++
SR-ATAD5	-
SR-HSE	+
SR-MMP	++
SR-p53	+++
**Toxicophore Rules**	
Acute Toxicity Rule	0 alert(s)
Genotoxic Carcinogenicity Rule	0 alert(s)
NonGenotoxic Carcinogenicity Rule	1 alert(s) DETAIL
Skin Sensitization Rule	5 alert(s) DETAIL
Aquatic Toxicity Rule	1 alert(s) DETAIL
NonBiodegradable Rule	2 alert(s) DETAIL
SureChEMBL Rule	0 alert(s)
FAF-Drugs4 Rule	1 alert(s) DETAIL

Tip: For the classification endpoints , the prediction probability values are transformed into six symbols: 0-0.1(---), 0.1-0.3(--), 0.3-0.5(-), 0.5-0.7(+), 0.7-0.9(++), and 0.9-1.0(+++).

Figure S44. The safety assessment data of **8q**.
